# Population genomics of *Culiseta melanura*, the principal vector of Eastern equine encephalitis virus in the United States

**DOI:** 10.1371/journal.pntd.0006698

**Published:** 2018-08-17

**Authors:** John Soghigian, Theodore G. Andreadis, Goudarz Molaei

**Affiliations:** 1 Department of Environmental Sciences, Center for Vector Biology & Zoonotic Diseases, The Connecticut Agricultural Experiment Station, New Haven, Connecticut, United States of America; 2 Department of Epidemiology of Microbial Diseases, Yale School of Public Health, New Haven, Connecticut, United States of America; University of Pittsburgh, UNITED STATES

## Abstract

**Background:**

Eastern Equine Encephalitis (EEE) (*Togaviridae*, *Alphavirus*) is a highly pathogenic mosquito-borne arbovirus that circulates in an enzootic cycle involving *Culiseta melanura* mosquitoes and wild Passeriformes birds in freshwater swamp habitats. Recently, the northeastern United States has experienced an intensification of virus activity with increased human involvement and northward expansion into new regions. In addition to its principal role in enzootic transmission of EEE virus among avian hosts, recent studies on the blood-feeding behavior of *Cs*. *melanura* throughout its geographic range suggest that this mosquito may also be involved in epizootic / epidemic transmission to equines and humans in certain locales. Variations in blood feeding behavior may be a function of host availability, environmental factors, and/or underlying genetic differences among regional populations. Despite the importance of *Cs*. *melanura* in transmission and maintenance of EEE virus, the genetics of this species remains largely unexplored.

**Methodology and principle findings:**

To investigate the occurrence of genetic variation in *Cs*. *melanura*, the genome of this mosquito vector was sequenced resulting in a draft genome assembly of 1.28 gigabases with a contig N50 of 93.36 kilobases. Populations of *Cs*. *melanura* from 10 EEE virus foci in the eastern North America were genotyped with double-digest RAD-seq. Following alignment of reads to the reference genome, variant calling, and filtering, 40,384 SNPs were retained for downstream analyses. Subsequent analyses revealed genetic differentiation between northern and southern populations of this mosquito species. Moreover, limited fine-scale population structure was detected throughout northeastern North America, suggesting local differentiation of populations but also a history of ancestral polymorphism or contemporary gene flow. Additionally, a genetically distinct cluster was identified predominantly at two northern sites.

**Conclusion and significance:**

This study elucidates the first evidence of fine-scale population structure in *Cs*. *melanura* throughout its eastern range and detects evidence of gene flow between populations in northeastern North America. This investigation provides the groundwork for examining the consequences of genetic variations in the populations of this mosquito species that could influence vector-host interactions and the risk of human and equine infection with EEE virus.

## Author summary

Eastern equine encephalitis (EEE) is a highly pathogenic mosquito-borne virus responsible for outbreaks of severe disease in humans and equines, causing high mortality and neurological impairment in most survivors. In the past, human disease outbreaks in the northeastern United States occurred intermittently; however, recently, this region has experienced a recurring seasonal intensification of EEE virus activity with expansion into more northerly locales. Eastern equine encephalitis virus is maintained in a transmission cycle involving the ornithophilic mosquito, *Culiseta melanura*, and wild passerine birds in freshwater swamp foci. Recent studies on the blood feeding behavior of *Cs*. *melanura* suggest this mosquito species could also be involved in transmission of EEE virus to humans and equines. Since variation in host feeding of this mosquito vector may be a function of environmental factors and/or genetic differences among regional populations, we examined the population genetics of *Cs*. *melanura* in order to: 1) characterize the genetic diversity of *Cs*. *melanura* at EEE virus foci across eastern North America, 2) investigate the occurrence of genetic structure among populations of *Cs*. *melanura*, and 3) examine patterns of gene flow among these populations. We generated a draft genome of this species as a reference for population studies, identified evidence of significant genetic differentiation and fine-scale genetic structure among populations, and found evidence of gene flow among northeastern populations. This study provides the molecular basis for future investigations on the causes and consequences of the genomic variation in *Cs*. *melanura* on the risk of human and equine infection with EEE virus.

## Introduction

Eastern equine encephalitis (EEE) virus (*Alphavirus*, *Togaviridae*) causes severe disease in humans and equines with high case mortality and persistent neurologic impairment in survivors [1]. Historically, outbreaks of this relatively rare but highly pathogenic arthropod-borne virus occurred intermittently in the eastern United States, predominantly in the mid-Atlantic and Gulf Coast states such as Florida, as well as in isolated foci in the northeast [2–6]. However, since the early 21^st^ century, this region has experienced a recurring seasonal intensification of EEE virus activity [5,7], and a northward geographic expansion [8–11]. In the northeastern United States, EEE virus is maintained in an enzootic transmission cycle in freshwater swamp foci involving the ornithophilic mosquito *Culiseta melanura* (Coquillett) (Diptera: Culicidae) and passerine birds [12–15]. Human and equine disease cases occur predominantly in close proximity to the freshwater swamp habitats in which *Cs*. *melanura* breeds [2,12].

*Culiseta melanura* is distributed throughout eastern North America [16] and is widely considered the principal enzootic vector of EEE virus. This mosquito species exhibits considerable variability in avian host choice across geographic regions, favoring Passeriformes birds [6,15,17–20]. In addition to avian hosts, recent studies indicate that between 1 and 11% of *Cs*. *melanura* bloodmeals originate from mammalian hosts including humans [6,17–20], suggesting the involvement of this mosquito species in epidemic/epizootic transmission of EEE virus to humans and equines [17,21]. Moreover, in a survey of thirty-five mosquito species from the northeastern United States, *Cs*. *melanur*a was the only species with the consistently high titers needed for transmission of the virus [21], and this species is among the predominant sources of virus isolations from field-collected mosquitoes in this region [21].

The apparent flexibility in host choice exhibited by *Cs*. *melanura* in various geographic regions [6,15,17–19] may be a function of environmental factors and/or underlying genetic variation that could influence vector-host interactions and potentially vectorial capacity. Despite the importance of *Cs*. *melanura* in transmission and maintenance of EEE virus, the genetics of this species remain largely unexplored. The present study was undertaken to gain insight into possible genetic variation in *Cs*. *melanura* populations that may contribute to its involvement in epidemic as well as enzootic transmission of EEE virus in certain locales. The specific objectives of the study were to: 1) characterize the genetic diversity of *Cs*. *melanura* in EEE virus foci across eastern North America, 2) investigate the occurrence of genetic structure among populations of *Cs*. *melanura*, and 3) examine patterns of gene flow among these populations.

## Methods

### *De novo* genome sequencing of *Culiseta melanura*

#### DNA isolation

Female *Culiseta melanura* from a laboratory colony, originally collected in Cape May, New Jersey [22] and maintained at the Connecticut Agricultural Experiment Station (CAES) since 2003, were used for DNA isolation. Briefly, an egg raft laid by a single female from this colony was isolated and her offspring were allowed to interbreed. Pupae from the fourth generation of this line were isolated and adults were frozen immediately upon emergence and held at -80°C. Adult females were pooled in a group of twelve and genomic DNA was extracted using the QIAGEN GenomicTip 10G kit (QIAGEN, Valencia, CA, USA), following manufacturer’s protocol, and using wide-bore tips for any transfer of genomic DNA.

#### Library preparation and sequencing

Extracted DNA was used for library construction and sequencing on the PacBio RSII instrument (Pacific Biosciences, Menlo Park, CA, USA) at the Yale Center for Genome Analysis (YCGA), where standard Pacific Biosciences 20Kb library construction was carried out according to the manufacturer’s instructions. The resulting library was loaded onto a total of forty SMRT Cells and sequenced with P6-P4 chemistry.

#### Assembly and polishing

Resulting raw reads were error-corrected and assembled using Canu version 1.6 [23]. Default settings were used with a genome size of 1.2 Gigabases; this genome size corresponded to initial size estimates suggested by an assembly of the first twenty SMRT Cells sequenced as well as previous estimates from cytophotometry [24]. The assembly was polished with the Pacific Biosciences Genomic Consensus suite, available in SMRT Analysis 5 [25] (publicly available at https://www.pacb.com/support/software-downloads/). Briefly, raw reads were mapped back to the genome assembly using BLASR, and high-quality consensus base calls were made with both Quiver and Arrow, resulting in two polished assemblies.

#### Assessment of genome completeness

Benchmarking Universal Single Copy Ortholog (BUSCO) Analysis [26] was used to assess the relative completeness of the draft genome assemblies before and after polishing, as well as to compare the polished genome to other publicly available vector genomes. The draft genome was queried to a curated catalog of 2799 putatively single copy ortholog genes common to Dipteran insects using BUSCO version 3 [27]. BUSCO catalogs represent expected single copy ortholog gene content for the taxonomic group in question. BUSCO retrieves complete, duplicated, and fragmented orthologs from the input genome based on this catalog and allows for a comparison of relative completeness among genomes. In addition to the draft genomes generated here, BUSCO analysis was performed on several publicly available Dipteran vector genomes from VectorBase (www.vectorbase.org, [28]) for comparison: *Aedes albopictus* (Foshan strain, Assembly AaloF1), *Anopheles gambiae* (PEST strain, AgamP4), *Culex quinquefasciatus* (Johannesburg strain, CpipJ2), *Glossina fuscipes* (IAEA strain, GfusI1), and *Lutzomyia longipalpis* (Jacobina strain, LlonJ1). BUSCO results were visualized in R with the ggplot2 package [29].

### Population genomics of *Culiseta melanura*

#### Sample collection

**Fig 1:**
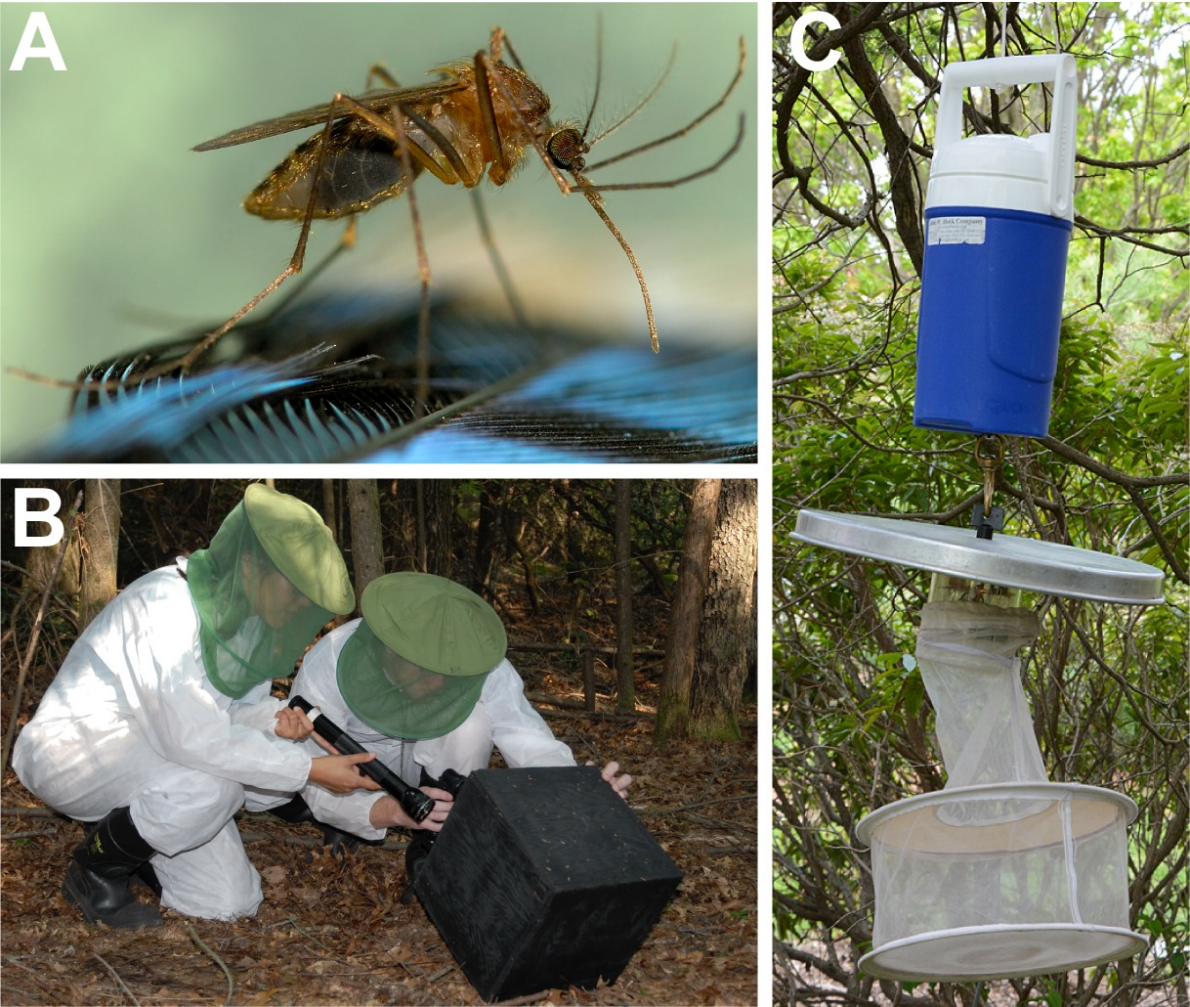
*Culiseta melanura* and two trapping methods used in this study. A) An engorged *Cs*. *melanura*. B) Collection of adult mosquitoes from a resting box. C) A CO_2_ baited CDC light trap used for collecting adult mosquitoes.

**Fig 2:**
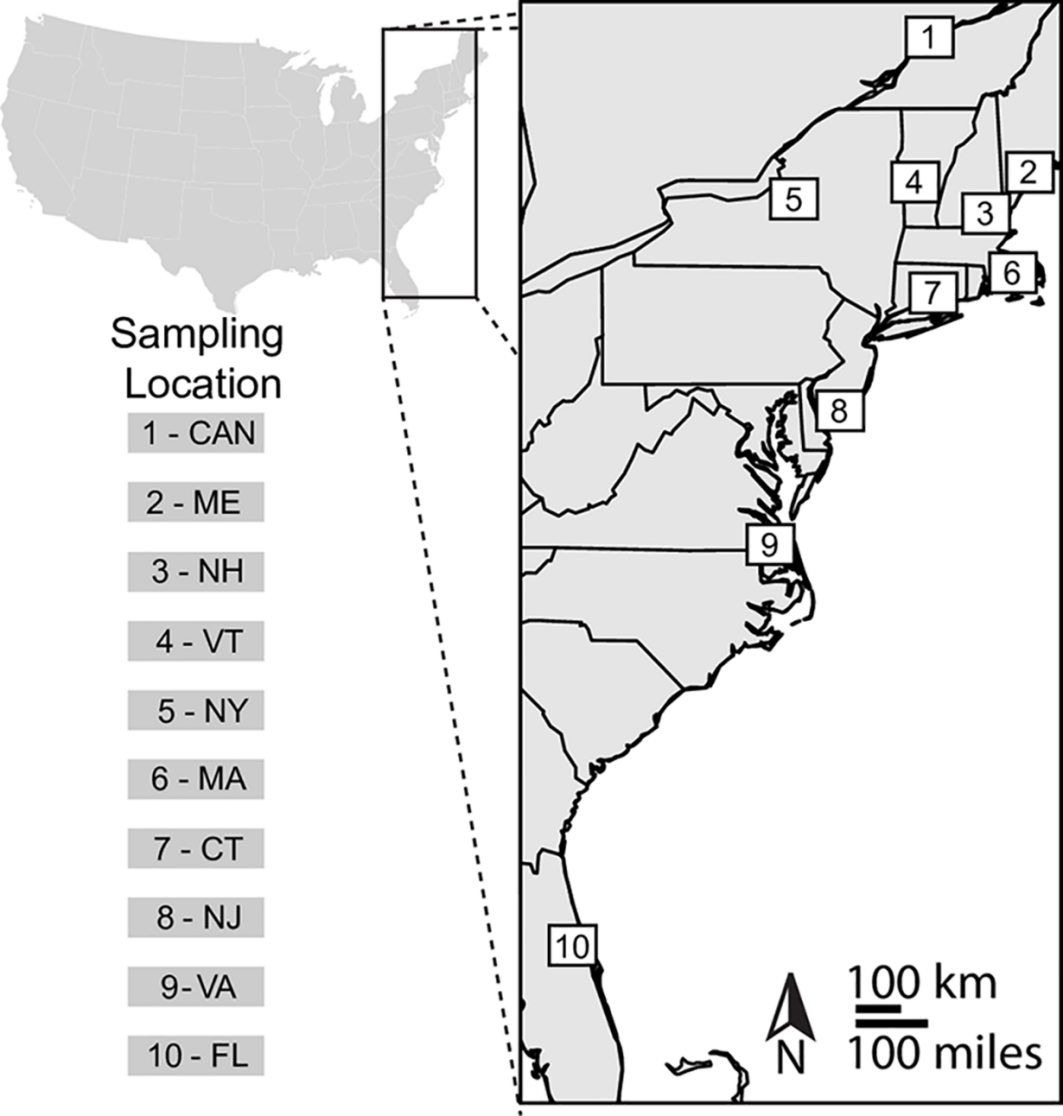
*Culiseta melanura* sampling sites from EEE virus foci across eastern North America. Additional information for each sampling site is given in Table 1. Map created using the package mapplots.

*Culiseta melanura* were collected from ten locations in eastern North America, ranging from Florida to Canada (Table 1, Figs 1 and 2). Locations fell approximately into three geographic regions: northern populations (1-CAN, 2-ME, 3-NH, 4-VT, 5-NY, 6-MA, 7-CT in Fig 2), a mid-Atlantic location in southern New Jersey (8-NJ in Fig 2), and southern populations (9-VA, 10-FL in Fig 2). Larvae were collected from Maine, Vermont, New Hampshire, and New Jersey using standard dipping method [30] and reared to adulthood, whereas female adults were sampled from the other six locations using CDC light traps with CO_2_, resting boxes, and nursery pots (Figs 1 and 2). Following collection, individuals were stored at -80°C for DNA extraction. Twelve females per population were individually homogenized with a pestle and DNA was extracted using a PureLink Genomic DNA Mini Kit (Invitrogen, Carlsbad, CA, USA) following manufacturer’s protocols. DNA was quantified using a Qubit Fluorometer (Invitrogen, Carlsbad, CA, USA) following extraction.

**Table 1:** Mosquito sampling sites from EEE virus foci across eastern North America.

ID	County/Admin. Region	State/Province	Region^a^	Library^b^
1-CAN	Montréal	Quebec, CAN	Northern	2
2-ME	York	Maine, USA	Northern	1
3-NH	Rockingham	New Hampshire, USA	Northern	3
4-VT	Rutland	Vermont, USA	Northern	5
5-NY	Oswego	New York, USA	Northern	3
6-MA	Plymouth	Massachusetts, USA	Northern	4
7-CT	Middlesex	Connecticut, USA	Northern	4
8-NJ	Cape May	New Jersey, USA	Mid-Atlantic	1
9-VA	Suffolk	Virginia, USA	Southern	2
10-FL	Volusia	Florida, USA	Southern	5

^a^ Region refers to the geographic region mentioned in the text (see Methods).

^b^ Library refers to locations that were pooled in the same sequencing lane (see Methods).

#### Adapter design and preparation

One of the most common criticisms of double-digest restriction size-associated DNA (ddRAD) methods [31–33] is the inability of the standard Peterson [31] protocol to differentiate PCR duplicates from multiple copies of an allele. In order to address this deficiency, a set of adaptors modified from Hoffberg et al. [34] was designed for the present study. Briefly, these adaptors include an oligo containing degenerate base pairs in the I5 index, in addition to the typical Read 1 and Read 2 adaptors common to ddRAD protocols that enable dual multiplexing of samples, and an Illumina index in the I7 position that allows multiplexing of libraries (Fig A in S1 File). The presence of degenerate base pairs in the I5 index allows for the detection of PCR clones, as true copies of an allele will have identical sequence reads, but will not share identical I5 indices, as this index is comprised of degenerate bases and is randomly annealed to fragments during library construction (see Library Construction, below).

The constituent oligo stubs that comprised Read 1 and Read 2 adaptors (Table A in S1 File) were annealed to form each respective adaptor by mixing 5 μl of 10 μM oligo stubs with the complimentary stubs, 5 μl of 10X NEB DNA Ligase Buffer (New England BioLabs, Ipswich, MA, USA), and 35 μl dH_2_0. Adaptor mixes were then heated at 97.5°C for 10 minutes, then cooled 2°C every minute to 4°C.

#### DNA digestion and library construction

Double-digest restriction site-associated DNA libraries were constructed from two populations at a time, in batches of twelve mosquitoes, with six individuals from each population per batch. Library construction was similar to the ‘optimized protocol’ of Hoffberg et al. [34], although pooling occurred prior to size selection, rather than after adaptor ligation. A total of 200 ng of genomic DNA from each sample was digested for 3 hours at 37°C in a 25 μl reaction with 1X CutSmart Buffer (New England Biolabs), 10 units of DdeI, and 10 units of MspI. These two common-cutting enzymes were chosen because they would yield many fragments compatible with the adaptor sets used in this study. Digestions were cleaned using AxyPrep Mag PCR Clean-up Kits (Axygen, Union City, CA, USA), hereafter magnetic beads, following manufacturers protocols.

Adaptors containing sample barcodes were then ligated to each individual sample in a 25 μl reaction with 17 μl of digested DNA, 2.5 μl of 10X T4 DNA Ligase Reaction Buffer (NEB), 1 μl of 10 μM read 1 adaptor, 2 μl of 10 μM read 2 adaptor, 1.5 μl of 10μM rATP (Promega, Madison, WI, USA), and 100 units of T4 DNA Ligase. Ligation reactions were incubated at room temperature for 30 minutes, then heat shocked at 65°C for 10 minutes, then cooled 2°C every minute to 4°C. The I5 index containing degenerate bases was then annealed to individually barcoded samples in a 50 μl reaction containing 22 μl of adapter-ligated sample, 2.5 μl of 10 μM I5 oligo, 0.3mM of each dNTP, 10 μl of 5X KAPA HiFi Fidelity Buffer, and 0.5 units of KAPA HiFi Hotstart DNA Polymerase (KAPA Biosystems, Wilmington, MA, USA). Samples were incubated at 95°C for 2 minutes, 98°C for 20 seconds, 61°C for 15 seconds, 72°C for 30 seconds, and 72°C for 5 minutes. Following incubation, samples were cleaned with magnetic beads. Next, samples were enriched in 50 μl reactions containing 22 μl of sample, 2.5 μl of 10 μM P5 primer, 2.5 μl of 10 μM I7 oligo containing either the “A” or “B” index (Table A in S1 File), 0.3mM of each dNTP, 10 μl of 5X KAPA HiFi Fidelity Buffer, and 0.5 units of KAPA HiFi Hotstart DNA Polymerase. Reactions were amplified in an 8 cycle PCR, following identical thermal cycling conditions as previously described.

Following enrichment, samples were cleaned a final time with magnetic beads, then quantified using a Qubit Fluorometer, and finally 175 ng of each sample was added to a pooled library of 24 total samples for size selection. Size selection of each library was performed on a BluePippin (Sage Science, Beverly, MA, USA) under a “tight” setting at 440 base pairs. Libraries were sequenced at the YCGA on the HiSeq 4000 platform (Illumina, San Diego, CA, USA) in 150 base pair paired-end mode. To improve sequence quality and increase the complexity of sequencing lanes, ddRAD libraries were spiked with another library.

#### Data processing

Resulting reads were demultiplexed initially by I7 index, and raw reads were contained in two paired end files (hereafter read 1 and read 2), as well as a third set of reads for the 8 degenerate bases in the I5 index in the appropriate phase for each paired end read. To remove PCR duplicates, the degenerate bases of the I5 index were concatenated to the end of read 1 using a custom script; and then, both paired end reads were passed to clone_filter in Stacks. With default settings, clone_filter removes reads that are exactly duplicate across both pairs of reads; as PCR duplicates would contain identical I5 indices (now concatenated to the end of read 1), as well as identical read 1 and read 2 sequences, they would be discarded by this filter. Following clone filtering, Trimmomatic [35] was used to remove the final 8 basepairs corresponding to the I5 index from each read 1 using the flag CROP. To confirm that the I5 index was properly removed following clone filtering, sequence length of FASTQ files was assessed prior to concatenation, after concatenation, and after cropping with Trimmomatic. Custom scripts used to concatenate FASTQ reads and assess length of sequences are available from the authors by request.

Following removal of PCR duplicates, Stacks version 1.46 [36,37] was used to demultiplex individual samples. Trimmomatic was then used to trim Illumina adaptors and quality filter reads, using a 4-base-pair sliding window and trimming where read quality dropped below 15. Trimmed reads were aligned to the Arrow-polished reference genome (see above) using Bowtie 2 version 1.2.2 in local mode [38]. The resulting alignments were used as input for the Stacks variant calling pipeline [36,37], requiring a minimum read depth of 5 to call a loci (flag -m 5 in pstacks). Next, variant calls were error corrected using the rxstacks module under a bounded SNP model, filtering biologically unrealistic haplotypes, and removing confounded loci [36]. In addition, reads were assembled *de novo* in Stacks using the subset strategy defined in Rochette and Catchen [37]; briefly, the six individuals per population with the highest coverage were used to construct a *de novo* Stacks catalog with parameters m and n equal to 5, and variants were called for all samples from this catalog.

Variants were called with Stacks’ population module, requiring that loci were present in 75% or more of samples (parameter r set to 0.75), and filtering minor alleles at a frequency below 0.01 (flag–min_maf 0.01). The effect of specifying the population parameter, p, was also evaluated by requiring that loci be present in 75% of samples in 2, 6, or 10 populations. For all analyses except for fineRADstructure analysis (see below), linked SNPs were filtered with PLINK version 1.9 [39] in a 50 base pair sliding window with the flag—indep-pairwise. PGDSpider version 2 [40] was used to convert between file formats.

#### Population differentiation and diversity

Several methods were used to assess genetic differentiation in *Cs*. *melanura* populations. Isolation by distance among sampling sites was assessed by correlating Nei’s genetic distance, implemented in the function dist.genpop from the R package adegenet [41], with geographic distance between sites. Significance of the correlation between genetic and geographic distance matrices was tested with a randomization-based Mantel test and 999 permutations, as implemented in the R package ade4 [42]. An analysis of molecular variance (AMOVA) [43] was used to detect population differentiation across sampling sites using the function stamppAmova from the R package StAMPP [44], considering both the case of separate sampling locations as populations, as well as by grouping sampling locations into northern (1-CAN, 2-ME, 3-NH, 4-VT, 5-NY, 6-MA, 7-CT in Fig 2) and southern (VA-9 and FL-10 in Fig 2) sets based on geographic sampling location. Pairwise genetic distance among sites was estimated by Weir and Cockerham’s Fst over 1000 permutations with the stamppFst function from StAMPP [44], and the mean pairwise Fst values within northern and southern populations was compared to the mean pairwise Fst values between these northern and southern populations, to evaluate whether populations were more genetically similar within geographic regions than between them.

Next, to assess the number of distinct genetic clusters, a least-squares approach was used: sparse non-negative matrix factorization (SMNF), implemented in the function smnf from the R [45] package LEA [46]. To choose the number of K clusters to visualize in the SMNF analysis, the mean cross entropy of each K value across 100 runs of smnf was visualized, and the K values with the lowest mean cross entropy were chosen. Then, from these K values, the lowest cross entropy run was selected for each K and the ancestry coefficients for all samples were visualized. In order to test if populations had significantly different ancestry to an unexpected genetic cluster (see Results), a one-way analysis of variance (ANOVA) with the R function aov was used with ancestry coefficients from the lowest cross entropy run for K = 2 as the dependent variable and sampling location as the independent variable.

FineRADStructure [47], a pipeline for the analysis of RAD data with the fineSTRUCTURE Markov chain Monte Carlo (MCMC) clustering algorithm [48], was used to observe coancestry among individuals and populations from the haplotypes output by Stacks. This pipeline leverages linkage information between loci to generate a nearest neighbor haplotype “coancestry” matrix from RAD data, which is then clustered with MCMC sampling to form closely related genetic clusters, where individuals sharing the highest degree of estimated relatedness are nearest to one another in a matrix. Additionally, this same pipeline was used to estimate relatedness of observed clusters with an ad hoc treebuilding approach, the maximum a posteriori state (MAP) tree [48].

Two multivariate methods were also used to visualize genetic structure across sampling locations: principal component analysis (PCA) and discriminant analysis of principal components (DAPC), both implemented in the R package adegenet [41,49]. A permutational multivariate analysis of variance (MANOVA) [50] was used to determine if populations differed significantly across the first five principal components from the PCA using the function adonis from the R package vegan [51,52] and 1000 permutations. Following a significant MANOVA, pairwise permutational MANOVAs were performed with the function pairwise.perm.manova and 1000 permutations from the package RVAideMemoire [53] to determine which populations were contributing significant variation to PCA components. Next, DAPC was used to determine if discriminant analysis could assign individuals back to their population of origin. To determine the number of principal components and discriminant functions to keep for DAPC and to avoid arbitrary overfitting, cross validation with 100 replicates was performed with the xvalDapc function, also from adegenet. Next, the genetic diversity of each population was estimated by assessing the number of private alleles and the mean heterozygosity of each population. The number of private alleles per population was estimated in R with the function private_alleles from the package poppr [54,55], and the mean heterozygosity per population was calculated in Arlequin version 3.5 [56].

Following the identification of an unexpected genetic cluster at sampling sites in the northeast (see Results below, Cluster A), additional analyses were performed. Briefly, to confirm morphological identification made prior to extraction, the ITS2 region from several mosquitoes used in this study was amplified using previously published PCR primers (ITS2-MOS-F and ITS2-MOS-R) [57]: all samples from 3-NH (3 in Fig 2), two from 3-ME (including one member of Cluster A; see below), and one mosquito chosen arbitrarily from 1-CAN, 4-VT, 8-NJ, 9-VA, and 10-FL. Reaction conditions consisted of 25 μl reactions containing 2 μl of sample, 1.25 μl of 10 μM ITS2-MOS-F primer, 1.25 μl of 10 μM ITS2-MOS-R primer, 0.3mM of each dNTP, 5 μl of 5X KAPA HiFi Fidelity Buffer, and 0.5 units of KAPA HiFi Hotstart DNA Polymerase. Samples were incubated at 95°C for 2 minutes, then 35 cycles of 98°C for 20 seconds, 61°C for 15 seconds, 72°C for 30 seconds, with a final extension step of 72°C for 5 minutes. Resulting PCRs were cleaned with magnetic beads and sequenced in both directions on a 3730xL DNA Analyzer (Applied Biosystems Inc., Grand Island, NY, USA) at the Keck Sequencing Facility, Yale University, New Haven, CT, USA. Sequences were assembled and aligned in Geneious version 9.1 with Geneious Alignment [58], and pairwise nucleotide identity was computed from this alignment.

R version 3.4 [45] was used for all analyses, except where noted. The R package mapplot [59] was used to create maps with polygons from this package’s world and state databases. The R package ggplot2 [29] was used to visualize results of population clustering and genetic diversity analyses, except for fineRADstructure analyses, where R scripts accompanying the pipeline were used instead.

## Results

### *Culiseta melanura* draft genome assembly

**Fig 3:**
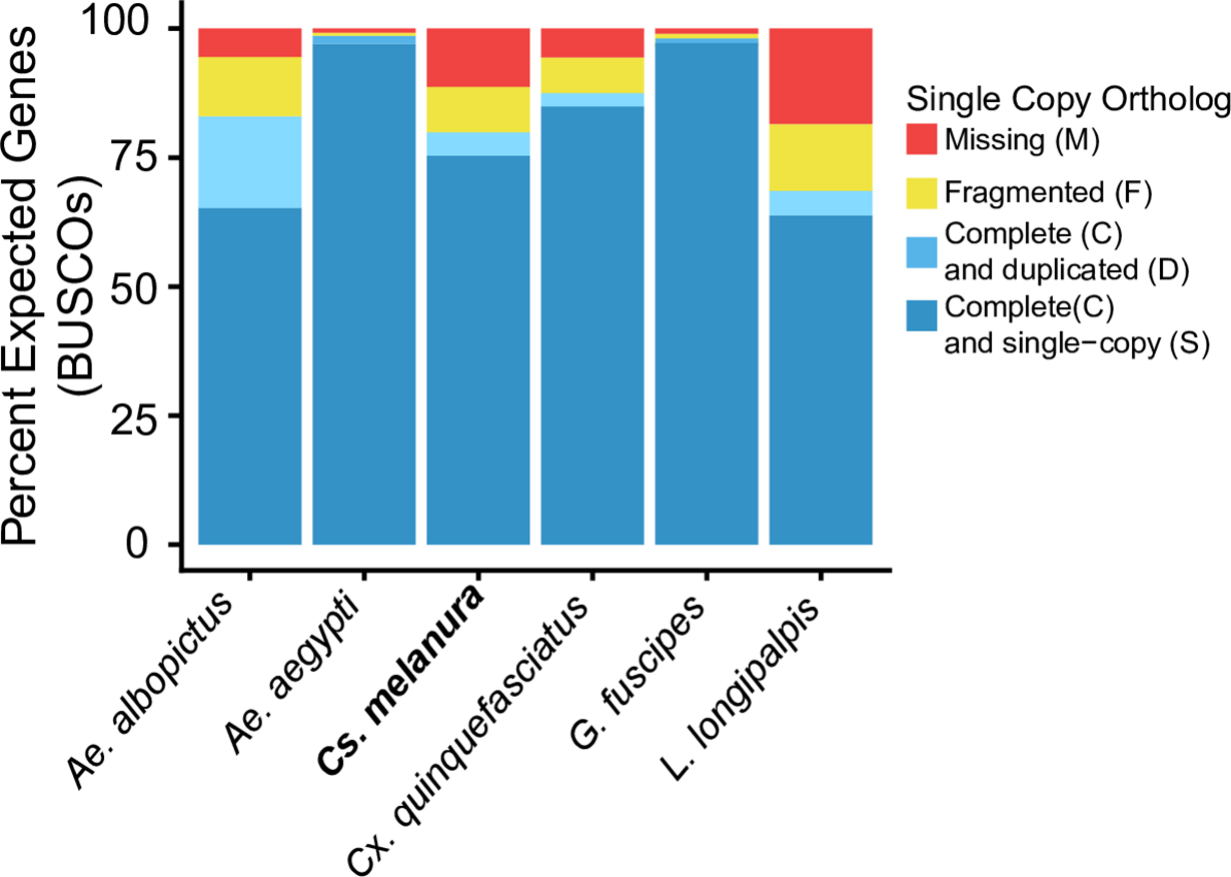
The BUSCO results for five publicly available Dipteran vector genomes, and the *Culiseta melanura* draft genome polished with Arrow. The draft genome generated in this study for *Culiseta melanura* has similar levels of completeness to several other vector genomes, with at least partial evidence of 88.7% single copy orthologs.

Sequencing of forty SMRT Cells yielded a total of 4.2 million raw reads with an average read length of 9 kilobases (kb). These raw reads were assembled to produce an unpolished draft assembly of 1.278 gigabases (Gb) across 25,500 contigs, with a contig N50 of 93.23 kb. Subsequent polishing provided modest increase to assembly size and contig N50. The Quiver-polished assembly was 1.279 Gb, with a contig N50 of 93.29 kb, while the Arrow-polished assembly was a total of 1.281 Gb, with a contig N50 of 93.36 kb. Although polishing provided only minor improvements to assembly size and N50, improvement to the results of BUSCO analyses were more substantial. The unpolished genome assembly had evidence of 61.3% of expected complete single copy orthologs, with 22.4% missing entirely. More than 70% of complete single copy orthologs were detected in both polished genomes, with the Arrow-polished assembly performing the best in BUSCO analyses; BUSCO results for this assembly found more than 75% of expected single copy orthologs, and in total, at least partial evidence for 88.7% of expected single copy orthologs (Full BUSCO Results: Complete, Single Copy: 75.4%; Complete, Duplicate: 4.5%; Fragmented: 8.8%; Missing: 11.3%; Fig B in S1 File). This Arrow-polished assembly performed similarity to other publicly available genomes on VectorBase (Fig 3).

### Polymerase chain reaction duplicates, alignment coverage, and variant detection

Polymerase chain reaction duplicates comprised 5.29% (SD = 4.0%) of reads across libraries. Of these, 71.9% (SD = 10.7%) were single duplications, although some reads were duplicated hundreds of times (Fig C in S1 File). Following removal of PCR duplicates and quality filtering, libraries resulted in 16.4 (SD = 4.3) million reads per mosquito, which aligned to the draft reference genome at a mean rate of 93.2% (SD = 3.8%). One mosquito from Canada had only 337,113 total reads and was removed from all downstream analyses, resulting in a total of 119 individuals from 10 geographic locations. Mean read depth across loci was 20.4X (SD = 5.8X) for the reference-aligned dataset, and 14.9X (SD = 4.3X) when Stacks was used for *de novo* assembly of loci (Table B in S1 File). In total, Stacks identified 3.9 million variant loci across all samples for the reference-aligned dataset, and 8.7 million variant loci for the *de novo* dataset.

Datasets varied in number of SNPs and genotyping rates (Table 2). Datasets in which p was set contained the largest number of SNPs, with more overall SNPs recovered as p declined, but with a lower genotyping rate. Fewer SNPs were recovered with Stacks’ *de novo* assembly of loci. The results presented below are for the variants called from the reference-aligned dataset, and without a specification of the population parameter p, which contained a total of 40,384 SNPs following the filtering of minor alleles and linked loci; specification of the population parameter to different values resulted in qualitatively similar results in downstream analyses, as did the *de novo*-assembled dataset (see Supporting Information for examples).

**Table 2:** Number of variants and genotyping rates for SNP datasets generated in this study.

Alignment Method	r^a^	p^a^	SNPs	Filtered SNPs^b^	Genotyping Rate
Reference-Aligned^c^	0.75	N/A	47163	40384	84% (SD = 7%)
Reference-Aligned	0.75	2	154921	81965	38% (SD = 8%)
Reference-Aligned	0.75	6	54855	43970	69% (SD = 12%
Reference-Aligned	0.75	10	23710	19099	87% (SD = 7%)
Stacks *de novo*	0.75	N/A	36026	19535	84%(SD = 11%)

^a^ The Stacks parameters controlling the proportion of individuals that must contain a locus (r) and the number of populations in which a locus must be present (p) for a locus to be included (see Methods).

^b^ The number of SNPs passing linkage filtering, and used in downstream analyses (see Methods).

^c^ The dataset from which results are presented in the main text.

### Population differentiation and genomic diversity

#### Population differentiation

There was no evidence of isolation by distance between sites (P>0.3; see Table C in S1 File). However, the AMOVAs found significant genetic variation among populations (p<0.001; Table D in S1 File) and between sets of northern and southern populations (p<0.001; Table D in S1 File). Subsequent pairwise Fst comparisons revealed small but significant Fst values among all populations of *Cs*. *melanura* sampled, ranging from 0.007 to 0.05 when excluding five mosquitoes belonging to an unexpected and highly divergent genetic cluster (Table E in S1 File; see below for details on these mosquitoes as Cluster A). In addition, the mean pairwise Fst value between northern populations (1-CAN, 2-ME, 3-NH, 4-VT, 5-NY, 6-MA, 7-CT in Fig 2) and southern populations (VA-9 and FL-10 in Fig 2) was 0.042, larger than mean pairwise Fst values within either geographic region (a mean Fst of 0.016 among northern populations, and a mean Fst of 0.015 among southern populations; Table E in S1 File).

#### Genetic clusters

The sparse non-negative matrix factorization (SNMF) analysis found evidence of multiple genetic clusters shared across datasets. For the reference-aligned dataset, regardless of the specification of the population parameter p, the K value with the lowest mean cross entropy was K = 2. However, because both K = 3 and K = 4 were close to K = 2 in cross entropy, all three values of K are presented here.

**Fig 4:**
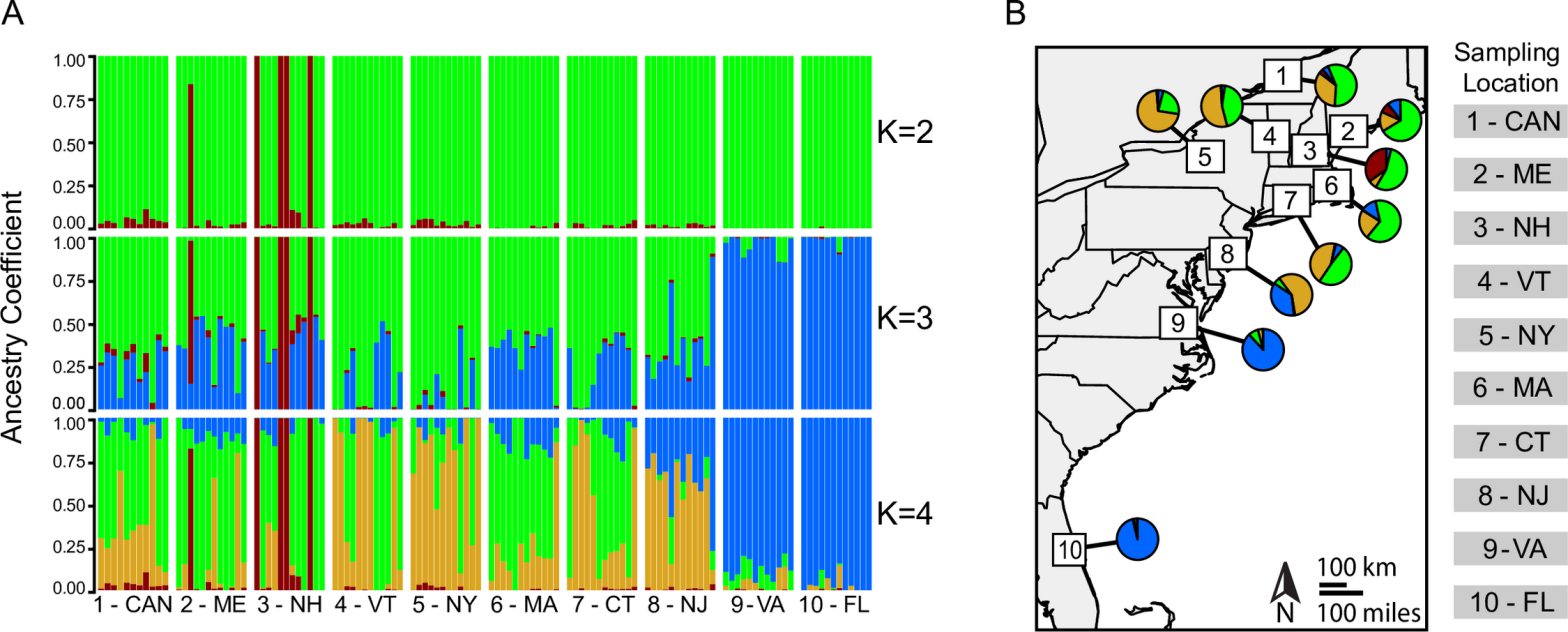
Results of SNMF genetic clustering analysis on 119 individuals from 10 populations of *Culiseta melanura*. (A) By individual, considering K = 2, K = 3, and K = 4. K = 2 had the lowest cross entropy, but both K = 3 and K = 4 were similar to K = 2 in cross entropy. (B) By population, with mean proportion of ancestry to each cluster for K = 4 represented as pie slices.

At all values of K investigated and regardless of dataset, an unexpected genetic cluster was detected by SNMF in the northeast. This cluster was comprised primarily of four individuals from New Hampshire and one from Maine, hereafter Cluster A (Fig 4, Fig D in S1 File). This genetic cluster was also detected in all other analyses performed (see below). Moreover, small degrees of ancestry (<15%) to this cluster were present across all northern sampling sites, and there was a significant difference among populations in proportion of ancestry from this cluster, even when the four mosquitoes from New Hampshire and one from Maine were excluded from other populations (one-way ANOVA, F_9,104_ = 4.68, p<0.001, Table F in S1 File). Of note, several northern populations, particularly 1-CAN and 5-NY had a relatively higher proportion of ancestry to Cluster A than did southern populations (Fig E in S1 File).

In addition to this unexpected genetic cluster, both datasets had substantial evidence for additional genetic clusters. Assuming K = 3 and K = 4, southern populations (9-VA, 10-FL) of this mosquito species formed a genetic cluster with >75% ancestry for all individuals (hereafter Cluster B, blue in Fig 4). At K = 3, an additional genetic cluster was detected, primarily associated with individuals from northern populations (1-CAN, 2-ME, 3-NH, 4-VT, 5-NY, 6-MA, 7-CT); and, assuming K = 4, these northern populations resolved into two genetic clusters (hereafter Cluster C, gold; and Cluster D, green; in Fig 4), the presence of which was also supported by additional analyses (see below). All northern populations (1-CAN, 2-ME, 3-NH, 4-VT, 5-NY, 6-MA, 7-CT) contained at least some individuals with ancestry to both Cluster C and Cluster D, indicating either admixture or ancestral polymorphism, but all northern populations also contained at least one individual with significant ancestry to each cluster (>75%), potentially indicating more recent gene flow or migration between Clusters C and D and among populations. Moreover, mosquitoes from New Jersey (8 in Figs 2 and 4), a population located geographically between northern (1-CAN, 2-ME, 3-NH, 4-VT, 5-NY, 6-MA, 7-CT) and southern populations (9-VA, 10-FL) showed mixed ancestry between Cluster B (associated predominantly with southern populations of 9-VA and 10-FL) and Cluster C (one of the northern-associated clusters). Hierarchical removal of genetic clusters (e.g. the removal of mosquitoes associated with Cluster A, or Cluster A and B, followed by a repeat of SMNF analysis) did not improve the resolution of SNMF-detected genetic clusters to the population level (Fig F in S1 File).

#### Clustering based on coancestry

**Fig 5:**
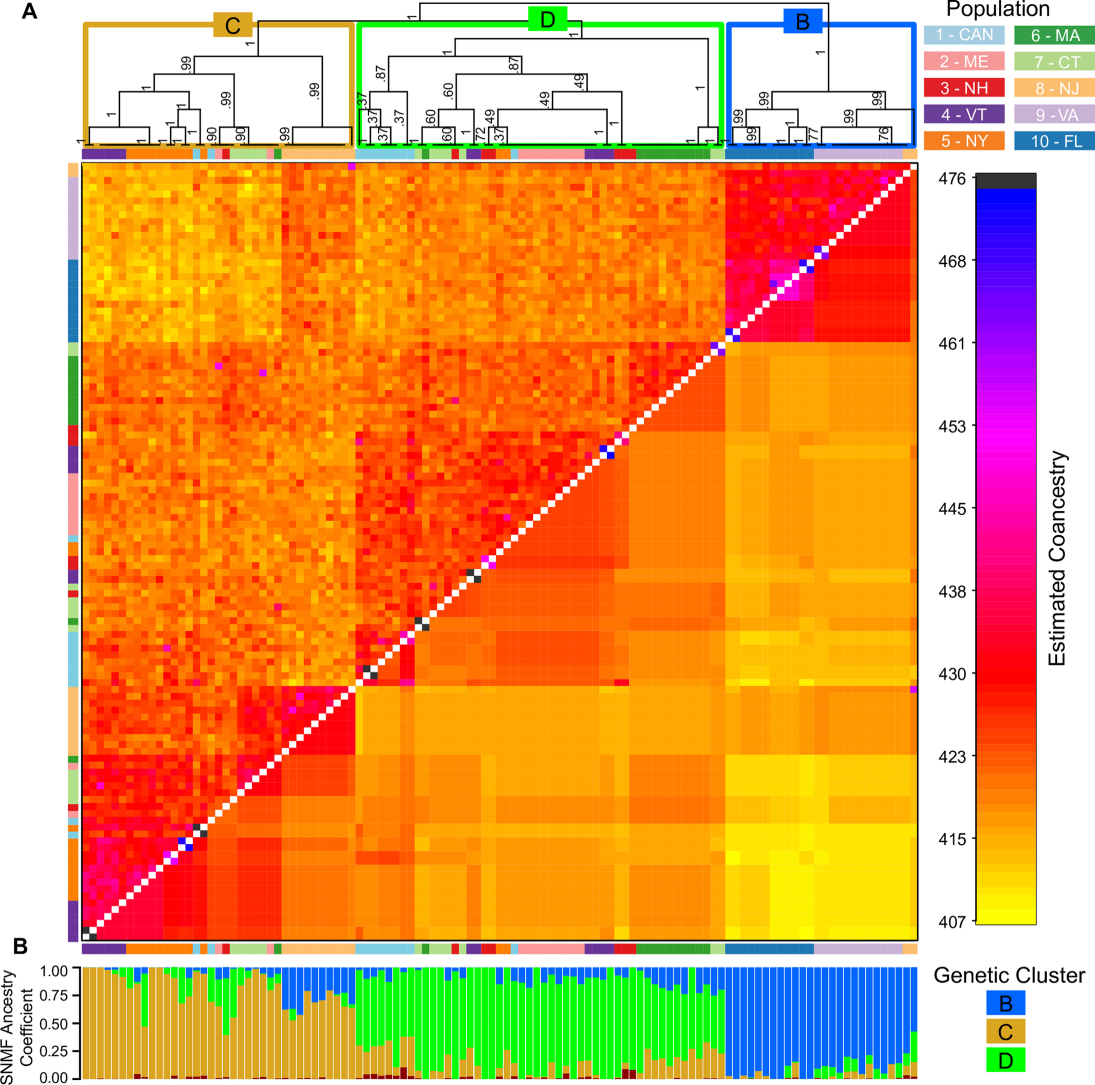
The fineRADstructure coancestry matrix and MAP tree. This analysis resolves the same genetic clusters as in other analyses and identifies fine-scale coancestry among populations and individuals. Cluster A was removed for this analysis to visualize finer scale structure between remaining clusters and populations (see Fig G in S1 File for the coanestry matrix containing Cluster A). A) In the coancestry matrix, individual coancestry is shown above the diagonal, while mean terminal clade coancestry is shown below the diagonal. Colored boxes over the MAP tree corresponds to the clusters in Fig 4 for K = 4. Posterior probability shown on branches. B) Results of the SNMF analysis for K = 4, ordered by coancestry.

Analyses with fineRADstructure recovered the same clusters as SNMF did at K = 4, with corresponding higher coancestry within clusters than among clusters, and high support values for clades comprised of each cluster in its maximum a posteriori state (MAP) tree (Fig G in S1 File). Cluster A resolved at the base of the MAP tree; Cluster B was sister to a clade consisting of Clusters C and D. This analysis also detected elevated coancestry levels between several northern individuals and Cluster A, particularly amongst samples from 1-CAN. After trimming of individuals associated with Cluster A (Fig 5), substantial substructure within the remaining genetic clusters was visible for most populations. Moreover, an examination of the coancestry matrix finds evidence that supports recent gene flow among populations, as some individuals share greater coancestry with a genetic cluster distinct from their sampling location. For instance, two individuals from 1-CAN (light blue in Fig 5A) have high coancestry to mosquitoes in Cluster C (and thus resolve within this cluster and a corresponding clade), despite the majority of mosquitoes from 1-CAN resolving within Cluster D (Fig 5A). This pattern is observed in several other populations (Fig 5A).

#### Multivariate analyses

Multivariate analyses showed a similar pattern to other analyses, indicating both regional differentiation and finer-scale structure among populations. Cluster A was markedly distinct from all other samples in the PCA (Fig H in S1 File). Following removal of Cluster A and the repetition of the PCA, the corresponding genetic clusters for B, C, and D were distinguishable (Fig H in S1 File). Although some populations were not easily distinguished visually in multivariate space (Fig H in S1 File), a permutational multivariate analysis of variance (MANOVA) using the first five principal components from this PCA as response variables and population of origin as the explanatory variable found a significant effect of population on variation in principal components (F_9,113_ = 12.3, R^2^ = 0.51, P<0.001). Subsequent pairwise randomization MANOVAs found significant differences among the majority of populations across the five principal components retained (Table G in S1 File), indicating underlying differences among populations across principal components.

**Fig 6:**
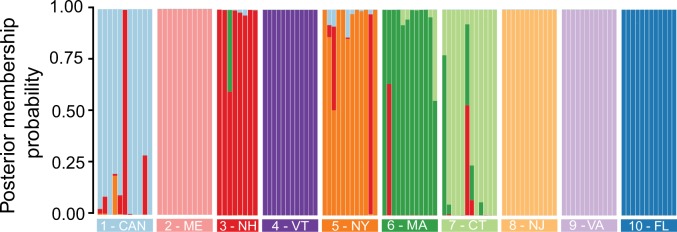
A genotype composition plot showing the posterior membership probability of mosquitoes following DAPC analysis. Individuals are grouped by the population. The majority of individuals are appropriately assigned to their respective populations.

Following PCA, discriminant analysis of principal components further resolved population structure across the majority of samples. Cross validation with 100 bootstrapped replicates identified 41 principal components as having the lowest root mean square error and the highest success rate of membership assignment. This analysis successfully assigned 95.6% of individuals (109/114) to their population from which they were sampled with greater than 50% membership probability (Fig 6), far outpacing the median of random assignment baseline success rate reported during cross validation, 9.84% (95% CI: ±0.05%). However, a small number of individuals from northern populations were improperly assigned, and individuals from five populations had at least partial posterior membership probability to other populations.

#### Heterozygosity and allelic diversity

Due to the degree of genetic differentiation detected in Cluster A, genomic diversity in these mosquitoes was assessed separately from their geographic sampling location and presented alongside statistics for each population. Mean heterozygosity was similarly low across all populations sampled (Table 3), with qualitatively higher heterozygosity amongst mosquitoes from Cluster A. In contrast, private alleles varied by population, despite a similar number of total alleles recovered across populations. Southern populations (9-VA, 10-FL) had the highest number of private alleles when considering geographic origin, followed by 8-NJ, a site geographically located between northern and southern sites in this study. Northern sites (1-CAN, 2-ME, 3-NH, 4-VT, 5-NY, 6-MA, 7-CT), excluding mosquitoes from Cluster A, had the lowest number of private alleles. Despite fewer alleles being recovered relative to other populations, Cluster A had 1820 private alleles, far more than any population (Table 3).

**Table 3:** Heterozygosity and allelic diversity in eastern populations of *Culiseta melanura*.

Population	N	H_O_	H_E_	Total Alleles	Private Alleles
1—CAN	11	0.20 (SD = 0.15)	0.23 (SD = 0.14)	58152	377
2—ME	11	0.19 (SD = 0.15)	0.22 (SD = 0.14)	60315	453
3—NH	8	0.23 (SD = 0.17)	0.27 (SD = 0.14)	55695	496
4—VT	12	0.19 (SD = 0.15)	0.22 (SD = 0.14)	60998	613
5—NY	12	0.19 (SD = 0.15)	0.22 (SD = 0.14)	60296	540
6—MA	12	0.19 (SD = 0.15)	0.22 (SD = 0.14)	59541	402
7—CT	12	0.18 (SD = 0.14)	0.21 (SD = 0.14)	60507	455
8—NJ	12	0.18 (SD = 0.14)	0.22 (SD = 0.14)	61239	955
9—VA	12	0.18 (SD = 0.15)	0.22 (SD = 0.14)	58880	981
10—FL	12	0.19 (SD = 0.15)	0.22 (SD = 0.14)	58998	1020
Cluster A	5	0.31 (SD = 0.21)	0.36 (SD = 0.13)	45790	1820

Mean observed heterozygosity (H_O_) and mean expected heterozygosity (H_E_) calculated based on the mean heterozygosity per loci per population. Total Alleles refer to the number of different alleles observed in a population.

#### Pairwise nucleotide identity of Cluster A from ITS2

Subsequent assays to confirm the identity of Cluster A using dye-terminator sequencing yielded approximately 300 bp of the ITS2 region from all individuals in Cluster A and several other mosquitoes from this study, all of which had been morphologically identified as *Cs*. *melanura* prior to DNA extraction for population genomics. The sequence alignment of *Cs*. *melanura* from this study resulted in pairwise nucleotide identity of >99% for all *Cs*. *melanura* samples, including individuals belonging to Cluster A (Table H in S1 File).

## Discussion

### *Culiseta melanura* draft genome

To facilitate the study of genetic variation in *Cs*. *melanura*, a draft genome was generated. This genome is comparable in presence of single copy orthologs to many other currently available assemblies of Dipteran vector genomes. The relatively large size of the *Cs*. *melanura* draft assembly, at over 1.24 Gb, is consistent with a previous estimate of the genome size of this species based on cytophotometry at 1.2 Gb [24]. Moreover, this is also consistent with the genome sizes of other mosquitoes in the subfamily Culicinae [24,60,61], such as *Aedes aegypti* where current genome assembly, AaegL5, is just under 1.28 Gb [28,61]. By comparison, the vectors of human malaria in the genus *Anopheles* have significantly smaller genomes: The current *Anopheles gambiae* assembly, AgamP4, is under 0.27 Gb. The draft assembly presented here was constructed principally as a reference for use in population studies of *Cs*. *melanura*, and further investigations are underway to improve the genome through additional sequencing and transcriptomics, a necessary first step to annotating the genome beyond the well-curated catalog of Dipteran single copy orthologs used in BUSCO analyses. As *Cs*. *melanura* is only the fourth non-Anopheline mosquito genome to be sequenced [60–62], further development of this genome could provide important insight into the ecology, evolution, and control of this and other vector species, as the availability of genomic data for vector species has enabled the characterization of pathways important to host seeking behavior (e.g. odorant receptors [63,64]), description of components of mosquito immunity [65–67], and the design of novel vector control methods [68–70].

The draft genome assembled in this study is a useful tool for population studies, as demonstrated here. Reads aligned to the reference at a mean rate greater than 93%, indicating the relative completeness as a reference. Utilization of the draft genome allowed the capture of a larger number of variants than under *de novo* variant calling with Stacks (Table 2), and with significantly less computation time. Thus, the draft assembly holds promise as a tool in future population studies, either as a reference for additional genotyping-by-sequencing, or for discovery of microsatellite markers, a low-cost alternative for the processing of larger sample sizes.

### Characteristics of the *Culiseta melanura* SNP dataset

The SNP dataset in this study recovered more than forty thousand markers passing filtering that were present in at least 75% of individuals. This dataset enabled the fine-scale differentiation of *Cs*. *melanura* populations (see Results, and below), despite the relatively small sample size. This is consistent with previous studies with empirical [71] and simulated [72] SNP data sets indicating that even sample sizes between two and ten [71–73] individuals per population can recover small degrees of significant genetic differentiation among populations, as long as at least 1500 SNPs are used [71]. Thus, the sample sizes used in this study should be adequate to evaluate even fine-scale genetic differentiation given the recovery of more than forty thousand SNPs. Moreover, this dataset provides an important framework for future studies that could expand on the sampling here, as RAD-seq datasets can be combined across laboratories with a high overlap of markers recovered, assuming identical library construction methods [74].

### Regional genetic differentiation and fine-scale population structure in *Culiseta melanura*

This study identified both regional differences between northern and southern populations of *Cs*. *melanura*, as well as fine scale population structure in this species. Pairwise Fst, AMOVA analyses, SNMF clusters, and PCA results all supported that populations within geographic regions of north (1-CAN, 2-ME, 3-NH, 4-VT, 5-NY, 6-MA, 7-CT) and south (9-VA and10-FL) were more akin to one another than to populations in other regions (Fig 4, Tables D and E in S1 File). These results suggest a degree of genetic differentiation at a geographic scale, although future studies are needed to confirm this differentiation and investigate its consequences with additional sampling, particularly in the southern United States, where our sampling was confined to two locations.

FineRADstructure identified finer-scale population structure than did the estimation of individual ancestry coefficients via the least squares-based method SNMF, consistent with results in other systems (e.g. with human SNP data [48]), as well as with simulations [47,48], indicating that the MCMC algorithm used by fineSTRUCTURE and fineRADstructure can be more sensitive than other clustering algorithms. The fine-scale population structure detected by fineRADstructure is consistent with the significant molecular variation and pairwise Fst values among populations, as well as the ability of DAPC to assign most individuals back to their correct population. Although fineRADstructure was unable to cluster individuals from three populations into single population clusters (3-NH, 4-VT, 7-CT), DAPC successfully assigned many of these individuals to their respective populations at a rate similar to several other northern sites, suggesting underlying genetic variation differentiating these populations.

The genetic variation among populations identified here could, among other factors, influence behavioral characteristics exhibited by *Cs*. *melanura* such as heterogeneities in host choice across regions [6,15,18,19], but correlating the fine-scale population structure reported here with previously observed host feeding patterns requires further investigations. Of note, human-derived blood meals from *Cs*. *melanura* have been identified in populations across the eastern United States, including from mosquitoes trapped near several locations utilized in this study, albeit at low frequencies (e.g. near 4-VT [17], 6-MA [19], 8-NJ [75], and 10-FL [18]). However, these populations belong to multiple genetic clusters identified in the present study, indicating that a propensity to feed on humans might be widespread across genetic clusters. Given the importance of this mosquito vector, the influence of population structure warrants further investigation, such as through the simultaneous sampling of engorged mosquitoes and SNP data across populations.

Moreover, our results are consistent with a post glacial recolonization of the northeast from southern populations following the retreat of the Laurentide ice sheet twelve to eighteen thousand years ago [76,77], and the melting of permafrost from the mid-Atlantic at this time period (e.g. near southern New Jersey [78]). The genetic cluster associated with southern populations (9-VA and 10-FL in Cluster B, Figs 4 and 5) is basal to Clusters C and D containing northern populations (1-CAN, 2-ME, 3-NH, 4-VT, 5-NY, 6-MA, 7-CT) and the mid-Atlantic population (8-NJ) in the MAP tree (Fig 5). Although the MAP tree is only an approximate guide to relatedness of populations [47,48], MAP trees perform well in simulations at inferring ancestral history between populations [48]. Moreover, southern populations (9-VA, 10-FL) had more private alleles than northern populations (1-CAN, 2-ME, 3-NH, 4-VT, 5-NY, 6-MA, 7-CT), indicating a higher genetic diversity (Table 3). The decline in private alleles south to north, as well as limited genetic variation among northern populations (e.g. the limited population structure, low pairwise Fst values, and lower number of private alleles), would be consistent with colonization northward and subsequent loss of genetic diversity through founder effects and/or bottlenecking events, the occurrence of which could be investigated in future studies. Thus, genomic evidence is consistent with the northward expansion of *Cs*. *melanura* from southern source populations. To confirm the pattern observed here, future studies with additional sampling throughout the range of this mosquito species could infer evolutionary history of these populations through Bayesian analyses estimating divergence time and biogeographic/demographic history [79,80].

### Gene flow among populations of *Culiseta melanura*

Substantial evidence of shared ancestry among populations was detected, likely indicating the presence of gene flow and/or shared ancestral polymorphism, particularly at the regional scales of northern (1-CAN, 2-ME, 3-NH, 4-VT, 5-NY, 6-MA, 7-CT) and southern (9-VA, 10-FL) populations. A lack of isolation by distance (Table C in S1 File), as well as lower pairwise Fst values among populations within a region compared to pairwise Fst values between northern and southern regions suggest not only a degree of differentiation between regions but could also indicate contemporary gene flow or a history of connectivity among populations [81,82]. At least one individual from northern sampling sites (1-CAN, 2-ME, 3-NH, 4-VT, 5-NY, 6-MA, 7-CT) had high ancestry with a different genetic cluster from the majority of those mosquitoes sampled at the same site, suggesting a degree of gene flow among genetic clusters (Fig 4). Moreover, the coancestry matrix and associated MAP tree provides additional evidence of gene flow among populations, as several individuals had higher coancestry with alternative genetic clusters and geographically distant populations in the north compared with other individuals from the same sampling location (e.g. two individuals from 1-CAN with higher coancestry to mosquitoes from 5-NY in Cluster C, Fig 5).

It is unlikely that this signal of gene flow is the result of direct dispersal among populations sampled in this study, as the mean flight range of *Cs*. *melanura*, estimated via mark and recapture, ranged from 4 to 9 kilometers [83]. Instead, these results suggest *Cs*. *melanura* in swamp habitats are not isolated at a regional scale, and there exists a large degree of connectivity among populations in northeastern North America.

### An unexpected genetic cluster of *Culiseta melanura*

In addition to the aforementioned fine-scale population structure, an unexpected genetic cluster was identified (Fig 4, Figs D, F, G in S1 File, Table E in S1 File). This cluster was comprised primarily of a small number of individuals from 2-ME and 3-NH; however, it also contributed substantial ancestry to individuals in other northern populations (particularly 1-CAN, 5-NY; Fig E in S1 File). As 2-ME and 3-NH were sequenced in separate sequencing lanes (Table 1) and had similar coverage and rates of alignment to the reference genome (Table B in S1 File) it seems unlikely this cluster is a sequencing artifact. Subsequent sequencing of ITS2 confirmed previous morphological identification that individuals belonging to Cluster A were indeed *Cs*. *melanura*, as pairwise identity of ITS2 sequences was >99% between individuals belonging to this cluster and all other *Cs*. *melanura* sequenced. Although the similarity in pairwise ITS2 sequence identity between members of Cluster A and other *Cs*. *melanura* may seem contrary to genomic results, this discrepancy is likely due to the greater number of variable sites in the SNP dataset, compared to only 297 nucleotide positions in the ITS2 alignment.

Mosquitoes belonging to Cluster A were relatively differentiated compared to mosquitoes belonging to other genetic clusters, with pairwise Fst values greatly exceeding those among other populations (Table E in S1 File). In addition, this cluster had far more private alleles than any population (Table 3). These results are consistent with evidence of an influx of genetic variation from a source not sampled in this study, such as admixture between northern populations of *Cs*. *melanura* and an unsampled and highly diverged population of this species, or interspecific hybridization with another closely related mosquito. However, due to the small number of individuals in this cluster, the lack of any previous evidence of hybridization between this species and others, and the paucity of sequencing information for other *Culiseta* species, it is not feasible with the available data to assess hypotheses regarding this cluster. Further studies investigating northeastern North American *Cs*. *melanura* populations with a larger sampling of mosquitoes, particularly targeting populations near 2-ME and 3-NH, may elucidate the origins of this cluster and any potential impacts it may have on behavioral characteristics of this mosquito species e.g. host feeding pattern and the risk of human infection with EEE virus.

### Conclusion

The results presented here provide the first insight into genetic variation in *Cs*. *melanura*. Although the existence of fine-scale structure and gene flow among populations was detected in these analyses, correlation between our findings and observed human and equine disease risk is difficult at this time. Future studies integrating sampling of populations along with mosquito bloodmeal analysis and observations of host diversity and abundance would be more informative to answering whether the genetic structure identified here is correlated with heterogeneities in blood host choice and/or disease risk across the region. By utilizing the new genomic resources available from this study, future investigations may build on the framework this study represents and provide greater insight into the vector-host interactions in *Cs*. *melanura* and its associated disease risk to humans and equines.

## Supporting information

S1 File The combined supporting tables and figures for this manuscript. This document contains Tables A-H and Figures A-H.
(DOCX)
