# Supplementary material for: Population genomics of Culiseta melanura, the principal vector of Eastern equine encephalitis virus in the United States
Source: PLoS Negl Trop Dis. 2018 Aug 17;12(8):e0006698. doi: 10.1371/journal.pntd.0006698 (PMC6114928; doi:10.1371/journal.pntd.0006698)
Supplement: S1 File — This document contains Tables A-H and Figures A-H. (DOCX) [file pntd.0006698.s001.docx]

Supporting Information File 1

This document contains the combined supporting material for Soghigian et al., *Population Genomics of Culiseta melanura, the Principal Vector of Eastern Equine Encephalitis Virus, in the United States*. Supporting Tables A through H are listed first, following by Supporting Figures A through H.

Supporting Tables

**Table A – The Oligo Sequences Used During Library Construction.**

| **Oligo Name** | **Sequence^a^** | **Complimentary Stub^b^** | **Usage** | **Note** |
| --- | --- | --- | --- | --- |
| P5 | AATGATACGGCGACCACCGA | N/A | Library enrichment | Used with I7 primers for library enrichment. |
| I5_N | AATGATACGGCGACCACCGAGATCTACAC ***NNNNNNNN*** ACACTCTTTCCCTA* C | N/A | Duplicate detection | Contains degenerate bases for PCR duplicate detection. |
| I7_A | CAAGCAGAAGACGGCATACGAGAT ***AGGTTCCT*** GTGACTGGAGTTCA*G | N/A | Library multiplexing | Used with P5 primer for library enrichment. |
| I7_B | CAAGCAGAAGACGGCATACGAGAT ***GCATTGGT*** GTGACTGGAGTTCA*G | N/A | Library multiplexing | Used with P5 primer for library enrichment. |
| R1_Stub_A | ACGACGCTCTTCCGATCT ***CCGAATAT*** | R1_Rcp_A | Sample Barcode (Read 1) | Forms Read 1 Adaptor A |
| R1_Rc_A | /5phos/CG ***ATATTCGG*** AGATCGGAAGAGCGTCGTGTAGGGAAAGAGTGT | R1_Stub_A | Sample Barcode (Read 1) | Forms Read 1 Adaptor A |
| R1_Stub_B | ACGACGCTCTTCCGATCT ***TTAGGCAAT*** | R1_Rc_B | Sample Barcode (Read 1) | Forms Read 1 Adaptor B |
| R1_Rc_B | /5phos/CG ***ATTGCCTAA*** AGATCGGAAGAGCGTCGTGTAGGGAAAGAGTGT | R1_Stub_B | Sample Barcode (Read 1) | Forms Read 1 Adaptor B |
| R1_Stub_C | ACGACGCTCTTCCGATCT ***GGTCTACGTAT*** | R1_Rc_C | Sample Barcode (Read 1) | Forms Read 1 Adaptor C |
| R1_Rc_C | /5phos/CG ***ATACGTAGACC*** AGATCGGAAGAGCGTCGTGTAGGGAAAGAGTGT | R1_Stub_C | Sample Barcode (Read 1) | Forms Read 1 Adaptor C |
| R2_Stub_1 | TNA ***ACGTTAG*** AGATCGGAAGAGCACACGTAATCC | R2_Rc_1 | Sample Barcode (Read 2) | Forms Read 2 Adaptor 1 |
| R2_Rc_1 | GTGACTGGAGTTCAGACGTGTGCTCTTCCGATCT ***CTAACGT*** | R2_Stub_1 | Sample Barcode (Read 2) | Forms Read 2 Adaptor 1 |
| R2_Stub_2 | TNA ***AGTACCGA*** AGATCGGAAGAGCACACGTAATCC | R2_Rc_2 | Sample Barcode (Read 2) | Forms Read 2 Adaptor 2 |
| R2_Rc_2 | GTGACTGGAGTTCAGACGTGTGCTCTTCCGATCT ***TCGGTACT*** | R2_Stub_2 | Sample Barcode (Read 2) | Forms Read 2 Adaptor 2 |
| R2_Stub_3 | TNA ***ACAACGATC*** AGATCGGAAGAGCACACGTAATCC | R2_Rc_3 | Sample Barcode (Read 2) | Forms Read 2 Adaptor 3 |
| R2_Rc_3 | GTGACTGGAGTTCAGACGTGTGCTCTTCCGATCT ***GATCGTTGT*** | R2_Stub_3 | Sample Barcode (Read 2) | Forms Read 2 Adaptor 3 |
| R2_Stub_4 | TNA ***AAGTGTAGCT*** AGATCGGAAGAGCACACGTAATCC | R2_Rc_4 | Sample Barcode (Read 2) | Forms Read 2 Adaptor 4 |
| R2_Rc_4 | GTGACTGGAGTTCAGACGTGTGCTCTTCCGATCT ***AGCTACACTT*** | R2_Stub_4 | Sample Barcode (Read 2) | Forms Read 2 Adaptor 4 |

a: Nucleotide sequences include special features added during primer creation, including phosphorothioate bonds (indicated with *) and 5’ phosphorylation. Barcodes are given as bold italics.

b: Complimentary stubs are annealed to one another as part of adapter construction.

**Table B.** **Comparison of the Reference-aligned Dataset and the *de novo* Dataset in Coverage.**

| **Site** | **Sample** | **Reference Coverage** | **Alignment Percent** | **De novo Coverage** | **Cluster A?** |
| --- | --- | --- | --- | --- | --- |
| CAN | 1 | 22 | 92.6 | 15 |  |
| CAN | 2 | 19 | 90.6 | 13 |  |
| CAN | 3 | 16 | 90.1 | 10 |  |
| CAN | 4 | 14 | 94.9 | 9 |  |
| CAN | 5 | 13 | 95.1 | 9 |  |
| CAN | 6 | 36 | 96.3 | 26 |  |
| CAN | 7 | 23 | 94.9 | 15 |  |
| CAN | 8 | 19 | 96.2 | 12 |  |
| CAN | 9 | 15 | 93.9 | 11 |  |
| CAN | 10 | 23 | 95.4 | 16 |  |
| CAN | 11 | 20 | 95.4 | 13 |  |
| FLA | 1 | 23 | 93.0 | 18 |  |
| FLA | 2 | 15 | 89.3 | 11 |  |
| FLA | 3 | 15 | 90.9 | 11 |  |
| FLA | 4 | 20 | 94.5 | 15 |  |
| FLA | 5 | 21 | 96.1 | 16 |  |
| FLA | 6 | 20 | 95.2 | 15 |  |
| FLA | 7 | 17 | 94.8 | 12 |  |
| FLA | 8 | 23 | 96.9 | 18 |  |
| FLA | 9 | 21 | 95.6 | 16 |  |
| FLA | 10 | 19 | 94.8 | 14 |  |
| FLA | 11 | 17 | 94.9 | 12 |  |
| FLA | 12 | 19 | 96.4 | 13 |  |
| CT | 1 | 21 | 91.4 | 16 |  |
| CT | 2 | 15 | 95.8 | 11 |  |
| CT | 3 | 15 | 92.1 | 12 |  |
| CT | 4 | 12 | 87.3 | 9 |  |
| CT | 5 | 13 | 91.8 | 10 |  |
| CT | 6 | 12 | 69.0 | 9 |  |
| CT | 7 | 12 | 84.6 | 8 |  |
| CT | 8 | 13 | 91.4 | 10 |  |
| CT | 9 | 12 | 89.8 | 8 |  |
| CT | 10 | 19 | 93.7 | 13 |  |
| CT | 11 | 20 | 94.5 | 15 |  |
| CT | 12 | 22 | 95.2 | 17 |  |
| MA | 1 | 13 | 88.9 | 10 |  |
| MA | 2 | 13 | 94.0 | 10 |  |
| MA | 3 | 14 | 89.1 | 11 |  |
| MA | 4 | 13 | 87.3 | 9 |  |
| MA | 5 | 13 | 89.2 | 10 |  |
| MA | 6 | 14 | 88.4 | 10 |  |
| MA | 7 | 14 | 87.4 | 10 |  |
| MA | 8 | 13 | 90.2 | 10 |  |
| MA | 9 | 13 | 92.5 | 9 |  |
| MA | 10 | 20 | 92.5 | 15 |  |
| MA | 11 | 20 | 95.7 | 15 |  |
| MA | 12 | 23 | 95.5 | 17 |  |
| ME | 24 | 21 | 89.7 | 15 |  |
| ME | 25 | 24 | 96.1 | 18 |  |
| **ME** | **26** | **21** | **94.9** | **16** | **Y** |
| ME | 27 | 17 | 87.9 | 13 |  |
| ME | 28 | 20 | 96.2 | 14 |  |
| ME | 29 | 20 | 95.9 | 14 |  |
| ME | 72 | 16 | 83.6 | 13 |  |
| ME | 75 | 18 | 94.0 | 13 |  |
| ME | 84 | 19 | 95.7 | 14 |  |
| ME | 86 | 25 | 92.5 | 18 |  |
| ME | 87 | 25 | 96.4 | 18 |  |
| ME | 90 | 21 | 96.4 | 15 |  |
| **NH** | **1** | **22** | **92.8** | **16** | **Y** |
| **NH** | **2** | **16** | **91.0** | **11** | **Y** |
| **NH** | **3** | **17** | **91.7** | **12** | **Y** |
| NH | 4 | 26 | 93.9 | 19 |  |
| NH | 5 | 18 | 95.5 | 13 |  |
| NH | 6 | 24 | 95.8 | 18 |  |
| **NH** | **7** | **21** | **95.2** | **15** | **Y** |
| NH | 8 | 31 | 96.2 | 23 |  |
| NH | 9 | 25 | 95.7 | 18 |  |
| NH | 10 | 22 | 95.7 | 16 |  |
| NH | 11 | 28 | 96.0 | 21 |  |
| NH | 12 | 22 | 96.0 | 16 |  |
| NJ | 72 | 25 | 95.2 | 19 |  |
| NJ | 75 | 22 | 94.5 | 15 |  |
| NJ | 84 | 20 | 86.3 | 17 |  |
| NJ | 86 | 27 | 96.6 | 16 |  |
| NJ | 87 | 26 | 93.3 | 19 |  |
| NJ | 90 | 27 | 95.8 | 15 |  |
| NJ | 24 | 20 | 94.5 | 14 |  |
| NJ | 25 | 23 | 95.9 | 20 |  |
| NJ | 26 | 25 | 95.6 | 19 |  |
| NJ | 27 | 19 | 94.9 | 12 |  |
| NJ | 28 | 16 | 89.3 | 14 |  |
| NJ | 29 | 18 | 89.8 | 21 |  |
| NY | 1 | 14 | 87.0 | 10 |  |
| NY | 2 | 24 | 85.3 | 17 |  |
| NY | 3 | 13 | 85.6 | 9 |  |
| NY | 4 | 27 | 94.7 | 20 |  |
| NY | 5 | 35 | 95.7 | 26 |  |
| NY | 6 | 14 | 91.9 | 10 |  |
| NY | 7 | 26 | 94.3 | 19 |  |
| NY | 8 | 17 | 95.5 | 12 |  |
| NY | 9 | 22 | 93.3 | 17 |  |
| NY | 10 | 13 | 92.6 | 9 |  |
| NY | 11 | 11 | 89.3 | 8 |  |
| NY | 12 | 25 | 95.8 | 19 |  |
| VA | 1 | 26 | 93.6 | 19 |  |
| VA | 2 | 26 | 94.3 | 19 |  |
| VA | 3 | 19 | 92.7 | 13 |  |
| VA | 4 | 34 | 95.0 | 24 |  |
| VA | 5 | 32 | 96.1 | 22 |  |
| VA | 6 | 32 | 96.0 | 23 |  |
| VA | 7 | 26 | 96.2 | 19 |  |
| VA | 8 | 35 | 96.5 | 25 |  |
| VA | 9 | 28 | 95.6 | 20 |  |
| VA | 10 | 29 | 95.2 | 21 |  |
| VA | 11 | 31 | 96.3 | 22 |  |
| VA | 12 | 31 | 95.5 | 21 |  |
| VT | 1 | 19 | 94.7 | 15 |  |
| VT | 2 | 14 | 90.5 | 10 |  |
| VT | 3 | 14 | 89.0 | 10 |  |
| VT | 4 | 24 | 95.1 | 18 |  |
| VT | 5 | 22 | 96.5 | 16 |  |
| VT | 6 | 24 | 94.6 | 19 |  |
| VT | 7 | 15 | 96.0 | 11 |  |
| VT | 8 | 21 | 96.8 | 16 |  |
| VT | 9 | 21 | 95.9 | 15 |  |
| VT | 10 | 23 | 95.2 | 18 |  |
| VT | 11 | 15 | 94.1 | 11 |  |
| VT | 12 | 23 | 96.4 | 18 |  |

Bold samples belong to Cluster A (See Results)

**Table C - Isolation by Distance in Populations of *Culiseta melanura*, Assessed with a Randomization Mantel Test.**

| **Populations Included** | **Observed correlation ( r )** | **p-value** |
| --- | --- | --- |
| All^a^ | 0.015 | 0.37 |
| Northern Only^b^ | 0.073 | 0.38 |

**^a^:** Excludes mosquitoes belonging to the highly-divergent Cluster A.

**^b^:** Includes populations 1-CAN, 2-ME, 3-NH, 4-VT, 5-NY, 6-MA, 7-CT in Fig. 2

**Table D –** **Analysis of Molecular Variance for Populations of *Culiseta melanura*.**

| **Populations Included** | **Sum of Square Deviations** | **Mean Square Deviations** | **Degrees of Freedom** | | **σ^2^** | **P-Value** |
| --- | --- | --- | --- | --- | --- | --- |
| All | 0.081 | 0.008 | | 10 | 0.001 | <0.001 |
| Error | 0.271 | 0.003 | | 108 | 0.003 |  |
| Total | 0.352 | 0.003 | | 118 |  |  |
|  |  |  | |  |  |  |
| N v S, NJ in N^a^ | 0.013 | 0.013 | | 1 | 0.0002 | <0.001 |
| Error | 0.299 | 0.003 | | 112 | 0.002 |  |
| Total | 0.313 | 0.003 | | 113 |  |  |
|  |  |  | |  |  |  |
| N v S, NJ in S^b^ | 0.012 | 0.012 | | 1 | 0.0002 | <0.001 |
| Error | 0.301 | 0.003 | | 112 | 0.003 |  |
| Total | 0.313 | 0.003 | | 113 |  |  |
|  |  |  | |  |  |  |
| N v S vs NJ^c^ | 0.019 | 0.009 | | 2 | 0.0002 | <0.001 |
| Error | 0.293 | 0.003 | | 111 | 0.003 |  |
| Total | 0.313 | 0.003 | | 113 |  |  |

**a**: The AMOVA with population assigned to geographic region as 1-CAN, 2-ME, 3-NH, 4-VT, 5-NY, 6-MA, 7-CT, 8-NJ and southern VA-9 and FL-10; see Fig. 2. The highly divergent Cluster A was removed for this analysis. See Results for details.

**b**: The AMOVA with population assigned to geographic region as 1-CAN, 2-ME, 3-NH, 4-VT, 5-NY, 6-MA, 7-CT and southern 8-NJ, VA-9, and FL-10; see Fig. 2. The highly divergent Cluster A was removed for this analysis. See Results for details.

**c**: The AMOVA with population assigned to geographic region as 1-CAN, 2-ME, 3-NH, 4-VT, 5-NY, 6-MA, 7-CT; southern as VA-9, and FL-10; and 8-NJ alone. The highly divergent Cluster A was removed for this analysis. See Results for details.

As 8-NJ appeared as admixed in subsequent analyses, molecular variance at the regional level was assessed with this population placed with northern populations, southern populations, or on its own. Results were qualitatively identical regardless of this.

**Table E –** **The Matrix of Pairwise Fst Values among Populations.^a^**

|  | **1 - CAN** | **2 - ME** | **3 - NH** | **4 - VT** | **5 - NY** | **6 - MA** | **7 - CT** | **8 - NJ** | **9 - VA** | **10 - FL** |
| --- | --- | --- | --- | --- | --- | --- | --- | --- | --- | --- |
| **2 - ME** | 0.011 |  |  |  |  |  |  |  |  |  |
| **3 - NH** | 0.020 | 0.011 |  |  |  |  |  |  |  |  |
| **4 - VT** | 0.010 | 0.012 | 0.023 |  |  |  |  |  |  |  |
| **5 - NY** | 0.013 | 0.019 | 0.029 | 0.007 |  |  |  |  |  |  |
| **6 - MA** | 0.019 | 0.012 | 0.018 | 0.016 | 0.024 |  |  |  |  |  |
| **7 - CT** | 0.011 | 0.010 | 0.020 | 0.009 | 0.012 | 0.007 |  |  |  |  |
| **8 - NJ** | 0.024 | 0.020 | 0.031 | 0.020 | 0.021 | 0.021 | 0.012 |  |  |  |
| **9 - VA** | 0.042 | 0.030 | 0.040 | 0.043 | 0.051 | 0.032 | 0.036 | 0.028 |  |  |
| **10 - FLA** | 0.051 | 0.038 | 0.046 | 0.049 | 0.058 | 0.039 | 0.044 | 0.038 | 0.015 |  |
| **Cluster A^b^** | 0.256 | 0.256 | 0.274 | 0.253 | 0.254 | 0.270 | 0.256 | 0.250 | 0.280 | 0.288 |

^a^ All pairwise Fst values are significantly different from zero (P<0.001).

^b^ Cluster A is comprised of four mosquitoes collected in New Hampshire and one from Maine. See Results for details.

**Table F:** **The One-Way Analysis of Variance on Ancestry Coefficients Across Populations for K=2.**

|  | **Degrees of freedom** | **Sum of Squares** | **Mean Squares** | **F-statistic** | **P-value** |
| --- | --- | --- | --- | --- | --- |
| Population | 9 | 0.01 | 1.4E-3 | 4.68 | 3.23E-05 |
| Residuals | 104 | 0.03 | 3E-4 |  |  |

**Table G.** **Pairwise MANOVA Results for the First 5 Principal Components of the PCA and Population of Origin.**

|  | 1 - CAN | 2 - ME | 3 - NH | 4 - VT | 5 - NY | 6 - MA | 7 - CT | 8 - NJ | 9 – VA |
| --- | --- | --- | --- | --- | --- | --- | --- | --- | --- |
| 1 - CAN |  |  |  |  |  |  |  |  |  |
| 2 - ME | 0.00 |  |  |  |  |  |  |  |  |
| 3 - NH | 0.02 | 0.00 |  |  |  |  |  |  |  |
| 4 - VT | **0.08** | 0.01 | 0.01 |  |  |  |  |  |  |
| 5 - NY | 0.02 | 0.00 | 0.00 | **0.31** |  |  |  |  |  |
| 6 - MA | 0.00 | 0.01 | 0.00 | 0.04 | 0.00 |  |  |  |  |
| 7 - CT | 0.02 | 0.01 | 0.00 | **0.07** | 0.02 | 0.11 |  |  |  |
| 8 - NJ | 0.00 | 0.00 | 0.00 | 0.00 | 0.00 | 0.00 | 0.00 |  |  |
| 9 - VA | 0.00 | 0.00 | 0.00 | 0.00 | 0.00 | 0.00 | 0.00 | 0.00 |  |
| 10 - FLA | 0.00 | 0.00 | 0.00 | 0.00 | 0.00 | 0.00 | 0.00 | 0.00 | 0.00 |

Bold P-values indicate non-significant test results. Pairwise tests are corrected for multiple comparisons, accounting for false discovery rate (FDR).

**Table H.** **Pairwise Nucleotide Identity of *Cs. melanura* from this Study.**

|  |  | *Cs. melanura ME26* | *Cs. melanura NH1* | *Cs. melanura NH2* | *Cs. melanura NH3* | *Cs. melanura NH7* | *Cs. melanura CAN3* | *Cs. melanura VT42* | *Cs. melanura FLA1* | *Cs. melanura VA1* | *Cs. melanura NH9* | *Cs. melanura NH4* | *Cs. melanura NH5* | *Cs. melanura NH6* | *Cs. melanura NH8* | *Cs. melanura NH10* | *Cs. melanura NH11* | *Cs. melanura NH12* | *Cs. melanura ME32* |
| --- | --- | --- | --- | --- | --- | --- | --- | --- | --- | --- | --- | --- | --- | --- | --- | --- | --- | --- | --- |
| *Cs. melanura* | ME26 |  | 99.83 | 99.83 | 99.83 | 99.83 | 99.83 | 99.50 | 99.83 | 99.50 | 99.82 | 99.83 | 99.50 | 99.50 | 99.50 | 99.83 | 99.83 | 99.83 | 99.82 |
| *Cs. melanura* | NH1 | 99.83 |  | 100.00 | 99.66 | 100.00 | 100.00 | 99.66 | 100.00 | 99.66 | 100.00 | 100.00 | 99.66 | 99.66 | 99.66 | 100.00 | 100.00 | 100.00 | 100.00 |
| *Cs. melanura* | NH2 | 99.83 | 100.00 |  | 99.66 | 100.00 | 100.00 | 99.66 | 100.00 | 99.66 | 100.00 | 100.00 | 99.66 | 99.66 | 99.66 | 100.00 | 100.00 | 100.00 | 100.00 |
| *Cs. melanura* | NH3 | 99.83 | 99.66 | 99.66 |  | 99.66 | 99.66 | 99.33 | 99.66 | 99.33 | 99.64 | 99.66 | 99.33 | 99.33 | 99.33 | 99.66 | 99.66 | 99.66 | 99.64 |
| *Cs. melanura* | NH7 | 99.83 | 100.00 | 100.00 | 99.66 |  | 100.00 | 99.66 | 100.00 | 99.66 | 100.00 | 100.00 | 99.66 | 99.66 | 99.66 | 100.00 | 100.00 | 100.00 | 100.00 |
| *Cs. melanura* | CAN3 | 99.83 | 100.00 | 100.00 | 99.66 | 100.00 |  | 99.66 | 100.00 | 99.66 | 100.00 | 100.00 | 99.66 | 99.66 | 99.66 | 100.00 | 100.00 | 100.00 | 100.00 |
| *Cs. melanura* | VT42 | 99.50 | 99.66 | 99.66 | 99.33 | 99.66 | 99.66 |  | 99.66 | 100.00 | 99.64 | 99.66 | 100.00 | 100.00 | 100.00 | 99.66 | 99.66 | 99.66 | 99.64 |
| *Cs. melanura* | FLA1 | 99.83 | 100.00 | 100.00 | 99.66 | 100.00 | 100.00 | 99.66 |  | 99.66 | 100.00 | 100.00 | 99.66 | 99.66 | 99.66 | 100.00 | 100.00 | 100.00 | 100.00 |
| *Cs. melanura* | VA1 | 99.50 | 99.66 | 99.66 | 99.33 | 99.66 | 99.66 | 100.00 | 99.66 |  | 99.64 | 99.66 | 100.00 | 100.00 | 100.00 | 99.66 | 99.66 | 99.66 | 99.64 |
| *Cs. melanura* | NH9 | 99.82 | 100.00 | 100.00 | 99.64 | 100.00 | 100.00 | 99.64 | 100.00 | 99.64 |  | 100.00 | 99.64 | 99.64 | 99.64 | 100.00 | 100.00 | 100.00 | 100.00 |
| *Cs. melanura* | NH4 | 99.83 | 100.00 | 100.00 | 99.66 | 100.00 | 100.00 | 99.66 | 100.00 | 99.66 | 100.00 |  | 99.66 | 99.66 | 99.66 | 100.00 | 100.00 | 100.00 | 100.00 |
| *Cs. melanura* | NH5 | 99.50 | 99.66 | 99.66 | 99.33 | 99.66 | 99.66 | 100.00 | 99.66 | 100.00 | 99.64 | 99.66 |  | 100.00 | 100.00 | 99.66 | 99.66 | 99.66 | 99.64 |
| *Cs. melanura* | NH6 | 99.50 | 99.66 | 99.66 | 99.33 | 99.66 | 99.66 | 100.00 | 99.66 | 100.00 | 99.64 | 99.66 | 100.00 |  | 100.00 | 99.66 | 99.66 | 99.66 | 99.64 |
| *Cs. melanura* | NH8 | 99.50 | 99.66 | 99.66 | 99.33 | 99.66 | 99.66 | 100.00 | 99.66 | 100.00 | 99.64 | 99.66 | 100.00 | 100.00 |  | 99.66 | 99.66 | 99.66 | 99.64 |
| *Cs. melanura* | NH10 | 99.83 | 100.00 | 100.00 | 99.66 | 100.00 | 100.00 | 99.66 | 100.00 | 99.66 | 100.00 | 100.00 | 99.66 | 99.66 | 99.66 |  | 100.00 | 100.00 | 100.00 |
| *Cs. melanura* | NH11 | 99.83 | 100.00 | 100.00 | 99.66 | 100.00 | 100.00 | 99.66 | 100.00 | 99.66 | 100.00 | 100.00 | 99.66 | 99.66 | 99.66 | 100.00 |  | 100.00 | 100.00 |
| *Cs. melanura* | NH12 | 99.83 | 100.00 | 100.00 | 99.66 | 100.00 | 100.00 | 99.66 | 100.00 | 99.66 | 100.00 | 100.00 | 99.66 | 99.66 | 99.66 | 100.00 | 100.00 |  | 100.00 |
| *Cs. melanura* | ME32 | 99.82 | 100.00 | 100.00 | 99.64 | 100.00 | 100.00 | 99.64 | 100.00 | 99.64 | 100.00 | 100.00 | 99.64 | 99.64 | 99.64 | 100.00 | 100.00 | 100.00 |  |
| *Cs. melanura* | NJ87 | 99.50 | 99.66 | 99.66 | 99.33 | 99.66 | 99.66 | 100.00 | 99.66 | 100.00 | 99.64 | 99.66 | 100.00 | 100.00 | 100.00 | 99.66 | 99.66 | 99.66 | 99.64 |

*Culiseta melanura* belonging to Cluster A are highlighted in gray.

Supporting Figures


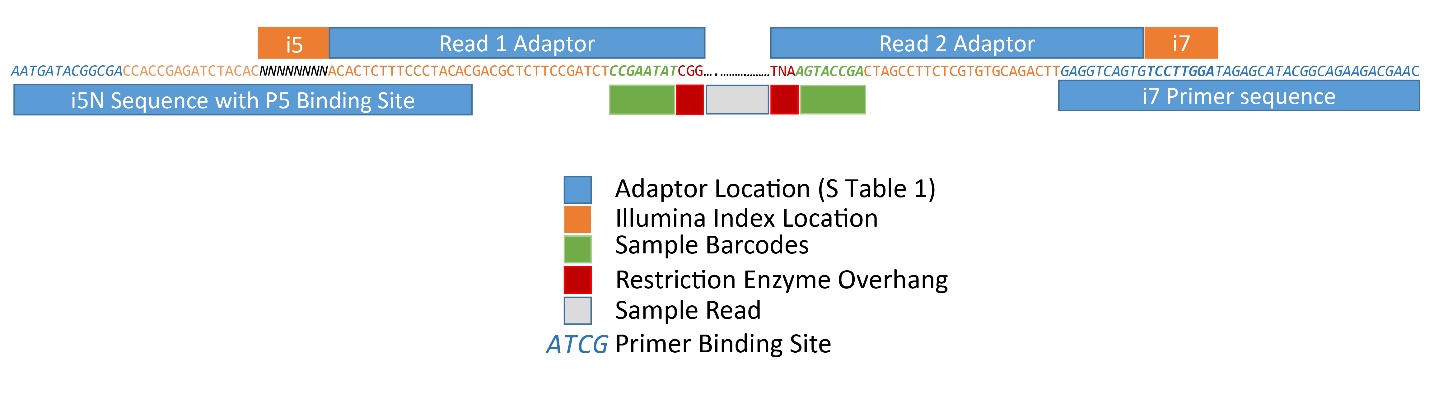


**Figure A.** **An Illustrated Version of the Annealed Adaptor Set Used in this Study**. The illustration includes the location of the degenerate I5 index, the I7 index, and sample barcodes for simple multiplexing. See Table S1 for complete sequences for adaptors.


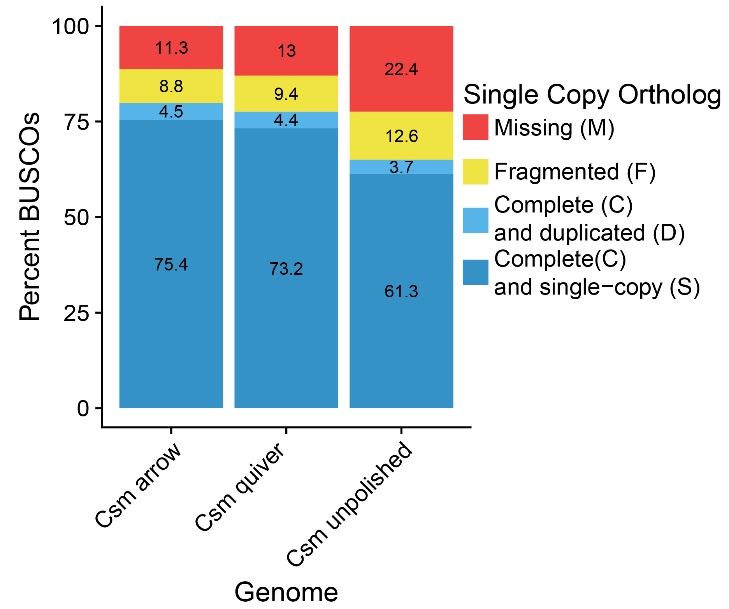


**Figure B.** **The BUSCO Results for the Raw Genome and the Quiver and Arrow-Polished Genomes.** Here, the Arrow-polished genome has the highest presence of single copy orthologs.


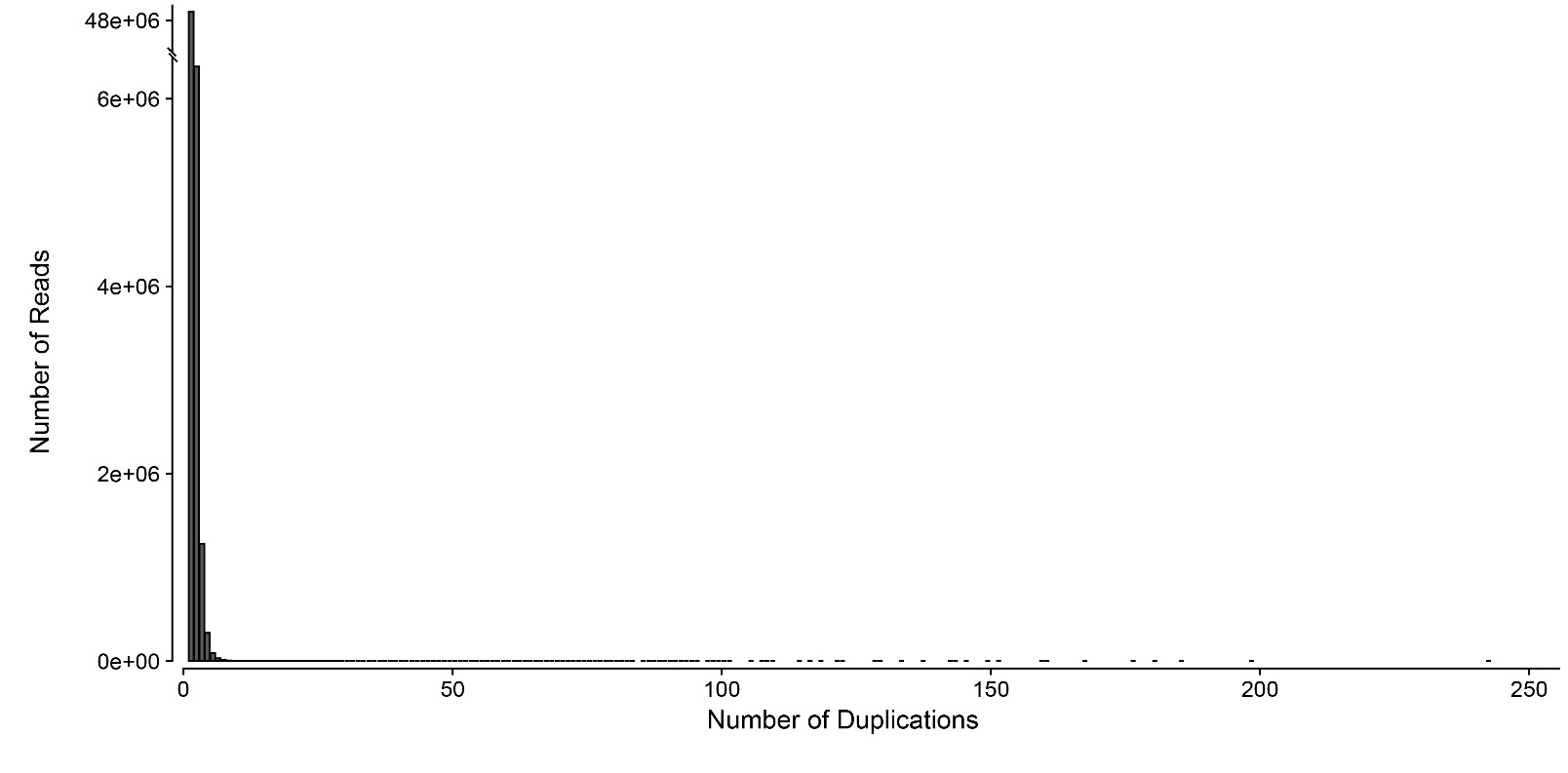


**Figure C**. **The Distribution of PCR Duplicates Across All Libraries.** The majority of duplicates were single copy duplicates, but some reads were duplicated >200 times.


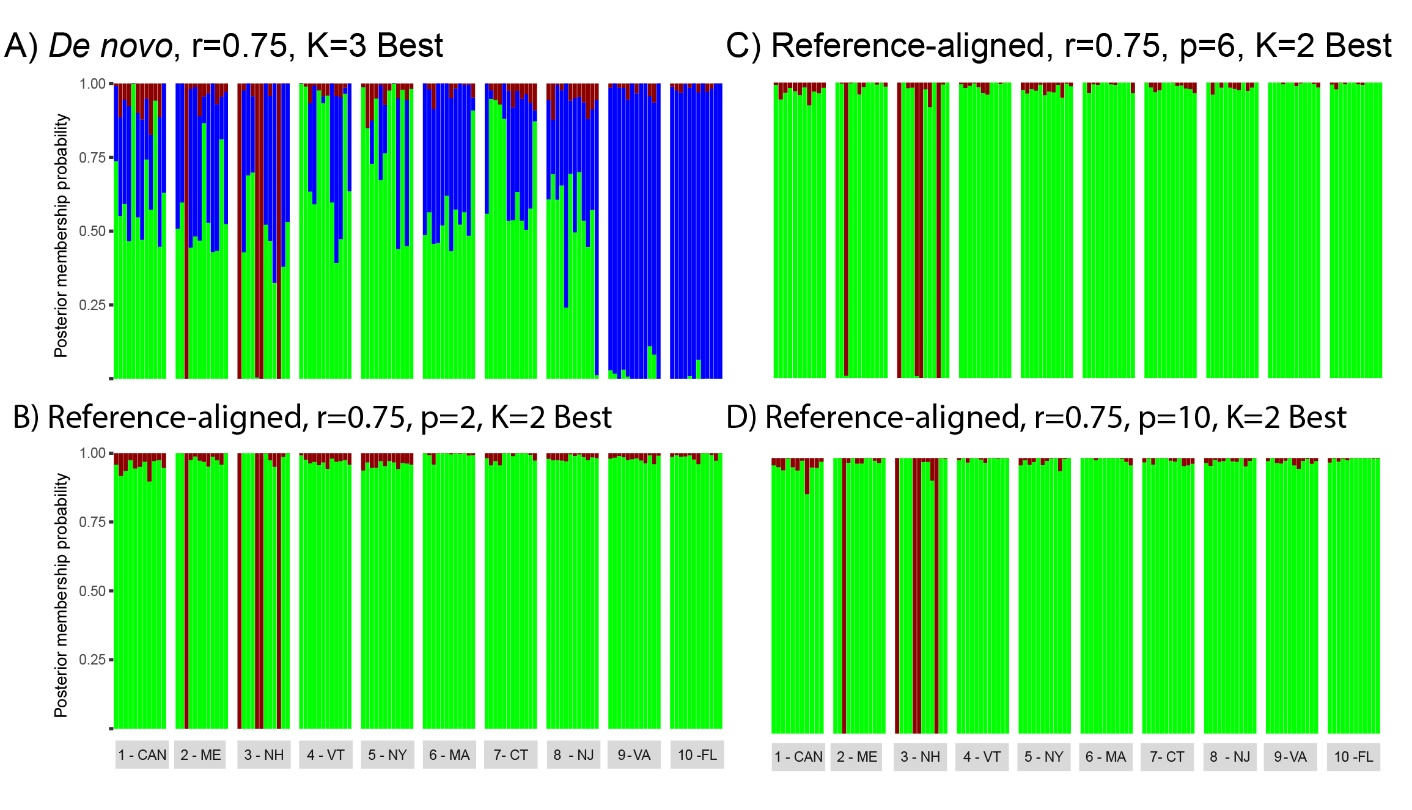


**Figure D**. **The SNMF Results for Alternative Datasets.** In all cases, an unexpected genetic cluster was present (in dark red). A) The *de novo* dataset, including only loci present in 75% of samples, where K=3 had the lowest cross entropy; B) The reference-aligned dataset, including only loci present in 75% of samples from at least 2 populations, where K=2 had the lowest cross entropy; C) As B, but from at least 4 populations; D) As B, but from at least 6 populations; E) As B, but from at least 10 populations.


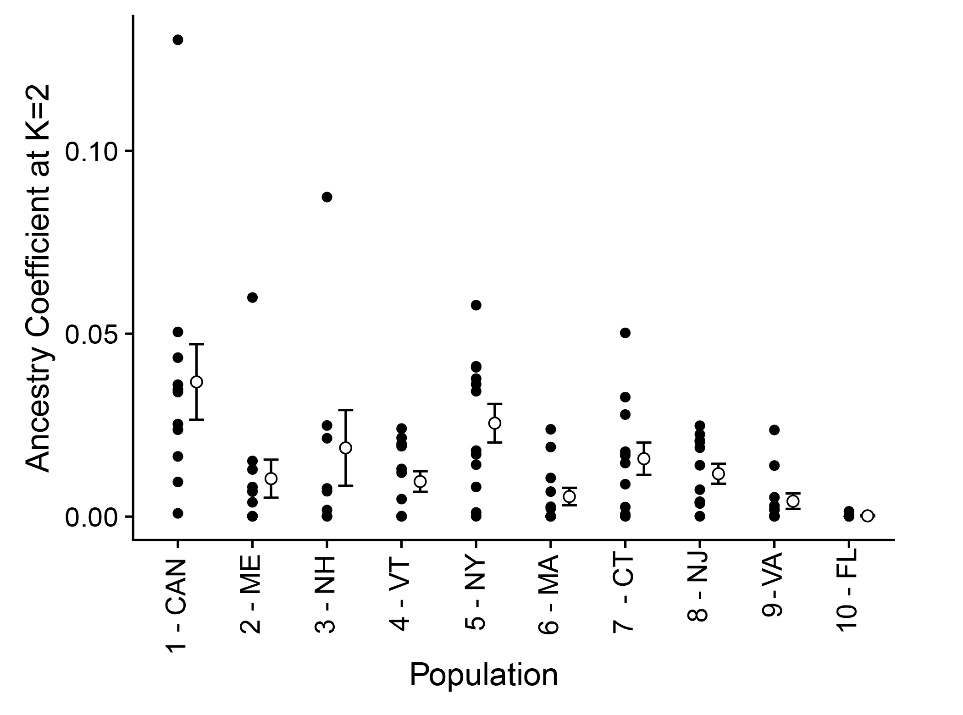


**Figure E**. **Mean Ancestry Coefficients for Each Population at K=2 with Cluster A Removed.** Closed circles are the ancestry coefficient values to Cluster A for each mosquito, while open circles are population means and error bars are standard errors of the mean.


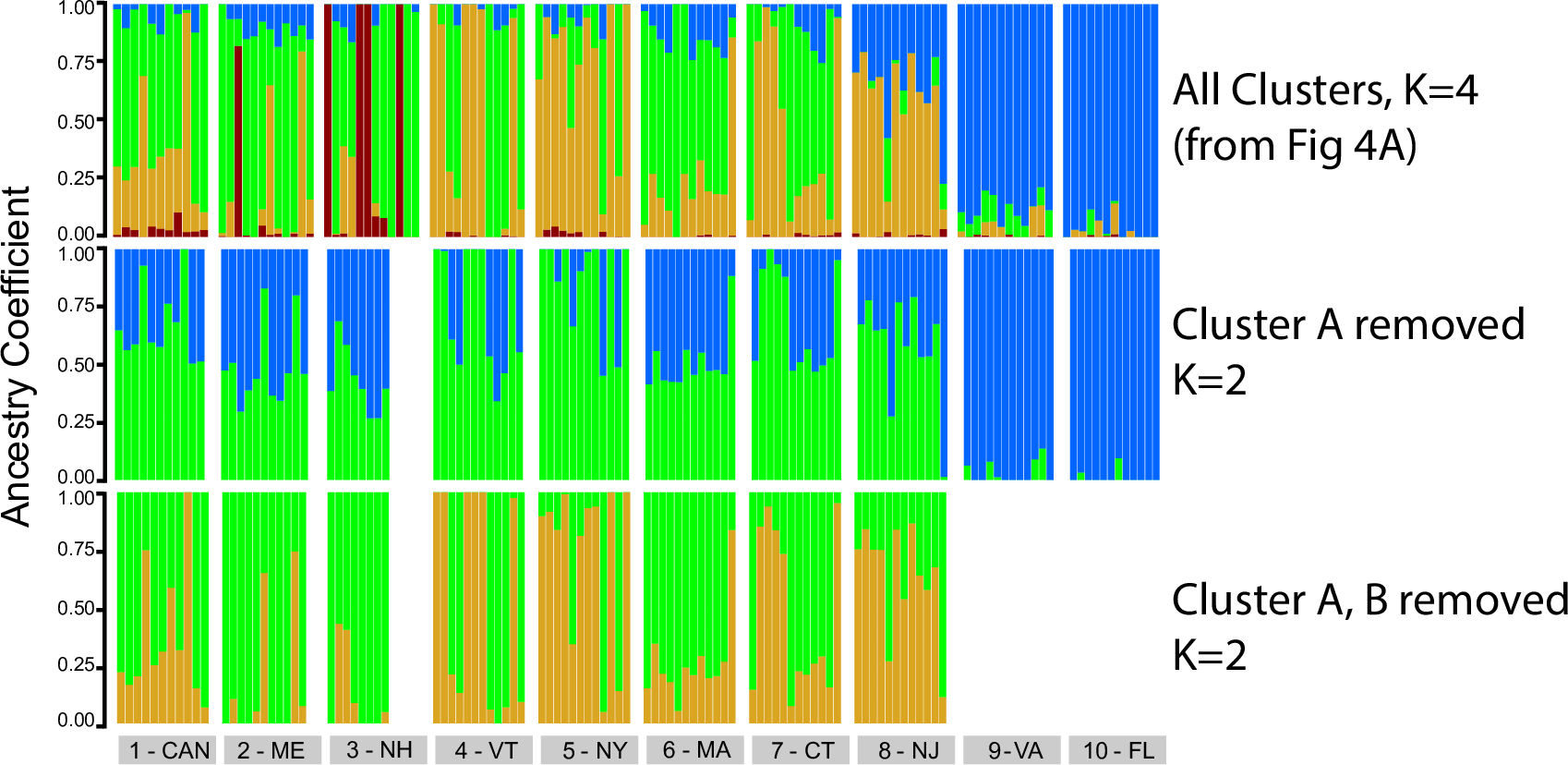


**Figure F. Hierarchical Removal of Clusters Does Not Improve Resolution of SNMF-detected Clusters.** The top panel, from Figure 4A, shows K=4 with all clusters included. Removal of Cluster A (Red) in middle, or Cluster B (Blue), bottom, does not improve the resolution of clusters to the population level.


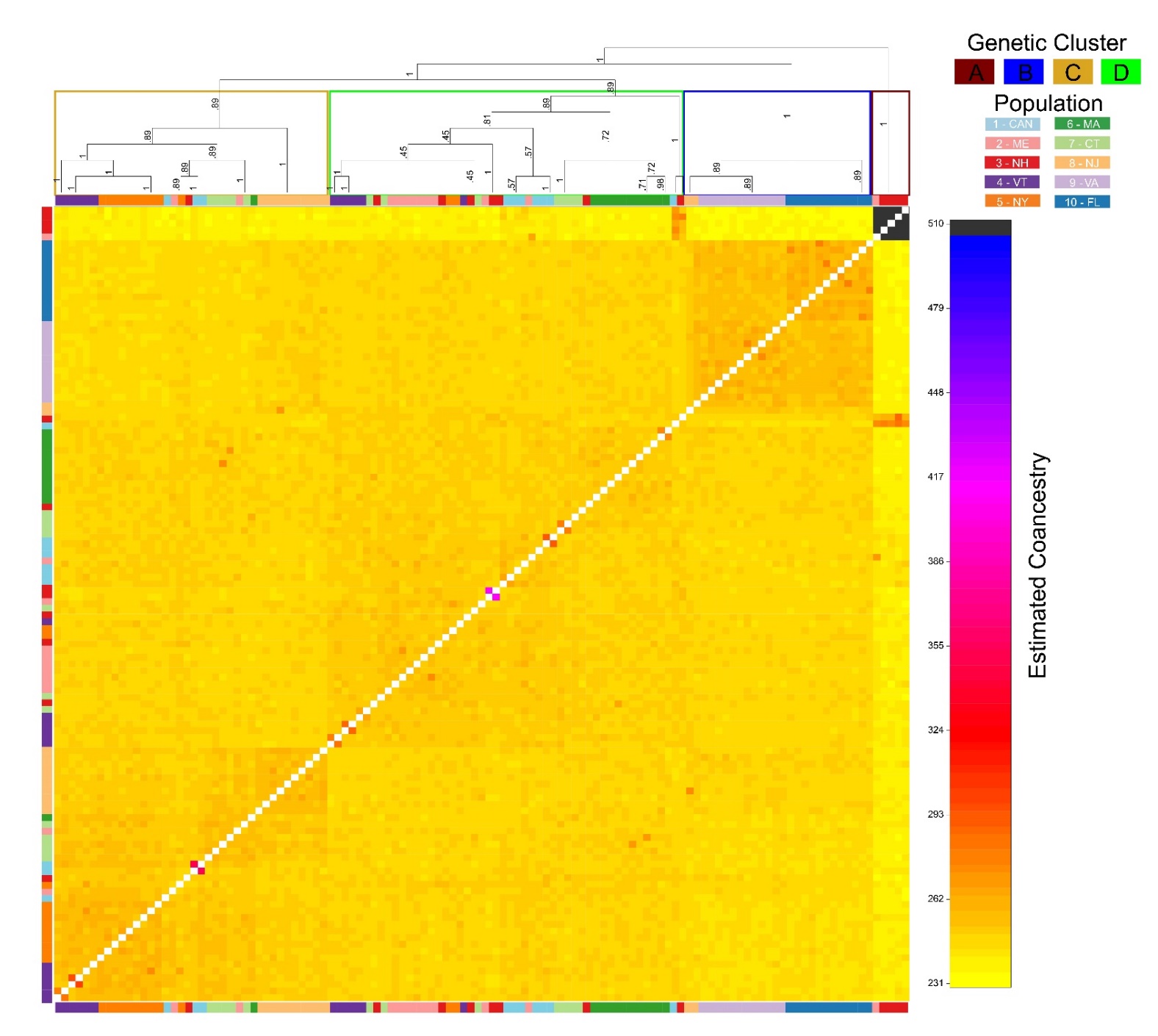


**Figure G**. **The Coancestry Matrix from fineRADstructure Prior to the Removal of Cluster A.** There is substantial coancestry between Cluster A and several northern individuals, particularly from Canada.

**
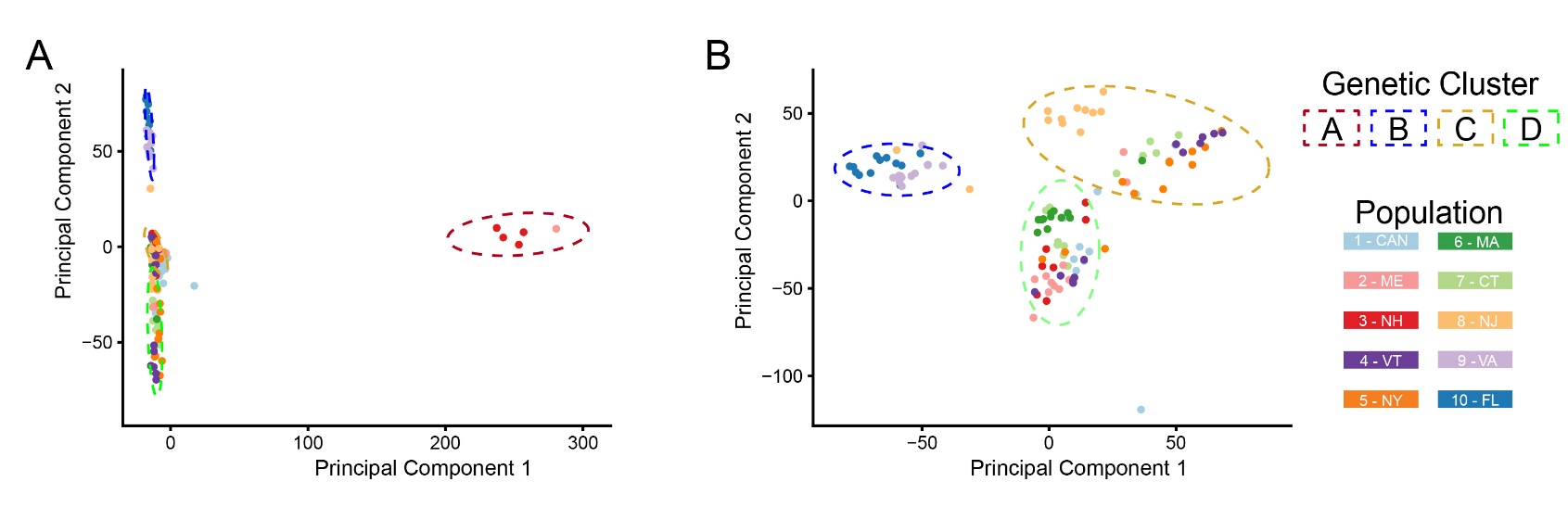
Figure H**. **The Results of Principal Component Analysis before (A) and after (B) the Removal of the Unexpected Genetic Cluster, Cluster A.** Principal components 1 and 2 shown for both PCAs, with 95% confidence ellipses drawn around individuals whose membership probability to a cluster exceeded 50% in Fig 4.
